# N-methyl-D-aspartate receptor inhibition protects against obesity-induced kidney disease

**DOI:** 10.1093/ndt/gfag027

**Published:** 2026-02-11

**Authors:** Àuria Eritja, Catalina Pérez-Olives, Dariel A Rodríguez Falcón, Maite Caus, Alicia García-Carrasco, Ana Martinez, Elias Jatem Escalante, Marisa Martin, Anton Jan van Zonneveld, Roel Bijkerk, Milica Bozic

**Affiliations:** Vascular and Renal Translational Research Group, Institut de Recerca Biomèdica de Lleida – Fundació Dr Pifarré, IRBLleida, Lleida, Spain; Vascular and Renal Translational Research Group, Institut de Recerca Biomèdica de Lleida – Fundació Dr Pifarré, IRBLleida, Lleida, Spain; Vascular and Renal Translational Research Group, Institut de Recerca Biomèdica de Lleida – Fundació Dr Pifarré, IRBLleida, Lleida, Spain; Vascular and Renal Translational Research Group, Institut de Recerca Biomèdica de Lleida – Fundació Dr Pifarré, IRBLleida, Lleida, Spain; Vascular and Renal Translational Research Group, Institut de Recerca Biomèdica de Lleida – Fundació Dr Pifarré, IRBLleida, Lleida, Spain; Vascular and Renal Translational Research Group, Institut de Recerca Biomèdica de Lleida – Fundació Dr Pifarré, IRBLleida, Lleida, Spain; Servicio de Nefrología, Hospital Arnau de Vilanova, Lleida, Spain; Servicio de Nefrología, Hospital Arnau de Vilanova, Lleida, Spain; Department of Internal Medicine (Nephrology) and the Einthoven Laboratory for Vascular and Regenerative Medicine, Leiden University Medical Centre, Leiden, The Netherlands; Department of Internal Medicine (Nephrology) and the Einthoven Laboratory for Vascular and Regenerative Medicine, Leiden University Medical Centre, Leiden, The Netherlands; Vascular and Renal Translational Research Group, Institut de Recerca Biomèdica de Lleida – Fundació Dr Pifarré, IRBLleida, Lleida, Spain

**Keywords:** chronic kidney disease, inflammation, lipotoxicity, NMDAR, obesity-induced kidney disease

## Abstract

**Background:**

Obesity-associated nephropathy is becoming increasingly prevalent, yet the molecular mechanisms linking metabolic stress to renal dysfunction remain unclear. N-methyl-D-aspartate receptors (NMDARs) are expressed in the kidney and are involved in the pathogenesis of different renal diseases. This study aimed to explore the role of NMDAR in obesity-induced kidney disease (OIKD).

**Methods:**

NMDAR expression was assessed in a murine model of OIKD, an *in vitro* fat-overload model, and in kidney biopsies from chronic kidney disease (CKD) patients. We combined pharmacological inhibition with gain- and loss-of-function strategies: NMDAR antagonists, memantine and MK-801, were tested *in vitro* and *in vivo*, while NMDAR was overexpressed and downregulated in cultured proximal tubular epithelial (HK-2) cells to evaluate its contribution to OIKD.

**Results:**

Free fatty acids (FFAs) exposure in HK-2 cells and high-fat diet (HFD) feeding in an OIKD mouse model led to increased GluN1, an essential subunit of NMDAR, accompanied by lipid accumulation and inflammatory responses associated with cellular injury. Kidney biopsies from CKD patients showed elevated GluN1 levels. *In vitro*, NMDAR antagonism attenuated FFA-induced cellular lipid accumulation and inflammation, regulated cellular redox homeostasis and suppressed FFA-induced p38 phosphorylation. GluN1 overexpression in HK-2 increased inflammatory markers, altered redox-regulating factors and promoted p38 activation. Treatment of cells with the p38 kinase inhibitor reduced FFA-induced GluN1 upregulation and attenuated inflammation. *In vivo*, NMDAR blockade reduced HFD-driven inflammation and tubular injury, and enhanced antioxidant gene expression.

**Conclusion:**

Our results demonstrate that renal NMDAR contributes to the pathogenesis of OIKD and underscore the therapeutic potential of targeting this receptor to attenuate inflammation and kidney injury in obesity-associated nephropathy.

KEY LEARNING POINTS **What was known:**

- Obesity is a major global health challenge and an independent risk factor for chronic kidney disease.
- Lipid accumulation, inflammation and oxidative stress drive renal injury, yet the precise molecular mechanisms underlying obesity-associated nephropathy remain unclear.
- N-methyl-D-aspartate receptors (NMDARs) have been implicated in renal disease, but their role in obesity-induced kidney injury is unknown.

**This study adds:**

- This study identifies NMDAR as a key mediator linking lipid overload to renal injury and inflammation.
- Inhibition of NMDAR attenuated inflammation, restored cellular redox homeostasis and reduced tubular injury *in vitro* and *in vivo*.
- NMDAR emerges as a novel therapeutic target in obesity-associated nephropathy.

**Potential impact:**

- Understanding the role of NMDAR in obesity-associated nephropathy may open new avenues for diagnosis and treatment of this disease.
- Targeting NMDAR offers potential for more effective therapies for metabolic kidney disease.

## INTRODUCTION

Obesity is a chronic, progressive condition that represents a major global health challenge. As reported by the World Health Organization, obesity has become an epidemic with an upward trend predicted for the coming years [1]. Obesity is also associated with an increased risk of chronic kidney disease (CKD) and its progression to end-stage renal disease [2–4]. Besides well-known factors that contribute to kidney injury in obesity, such as obesity-related hyperfiltration, altered adipokine and cytokine signaling, and activation of the renin–angiotensin–aldosterone system [5], disturbances in fatty acid and cholesterol metabolism lead to lipid accumulation and nephrotoxicity, with both plasma and intrarenal lipid accumulation contributing to structural and functional changes in the kidney [6–9]. Excessive lipid deposition in cells unable to manage high lipid loads, particularly proximal tubular epithelial cells [10], can trigger tissue inflammation [7, 11] and oxidative stress [12], key mechanisms underlying kidney damage, and subsequently lead to the progression of glomerulosclerosis and tubulointerstitial fibrosis [7, 9, 13]. Despite increasing reports linking obesity to CKD, the exact mechanism leading to the onset of kidney injury and dysfunction in obesity-associated nephropathy remains unclear.

N-methyl-D-aspartate receptor (NMDAR) is a non-selective cation channel and a member of a heterogeneous family of ionotropic glutamate receptors, with specific functional and pharmacological properties [14, 15]. It typically functions as a tetramer of two GluN1 and two GluN2 subunits [16, 17], both essential for the formation of a functional channel [14, 18]. NMDAR is expressed in the central nervous system (CNS) [14, 19] and peripheral tissues, including the kidney [20–22], where it plays important roles in renal vasodilation, proximal tubule reabsorption and glomerular filtration [20, 23, 24], as well as in the maintenance of the epithelial phenotype of proximal tubular cells [22]. Dysregulation of NMDAR has been implicated in diabetic nephropathy [25], ischemic kidney injury [26], secondary hyperparathyroidism in CKD [27] and glomerular disorders [20]. Thus, glutamatergic signalling via NMDAR plays an important role within the kidney, and its effects are strongly dependable on the subtle balance between the activation and the blockade of the receptor.

The NMDAR is a complex protein assembly with multiple binding sites regulated by endogenous and exogenous factors [14]. Among these, lipids modulate NMDARs, while cholesterol and its metabolites significantly affect its expression in the CNS [28, 29]. However, the role of NMDAR in obesity-induced kidney disease (OIKD) remains unknown.

The aim of this study was to explore the role of NMDAR in OIKD using integrated experimental approaches in renal tubular epithelial cells and mouse model of OIKD. Our findings may help identify a novel therapeutic strategy to mitigate obesity-associated nephropathy and improve kidney health in metabolic disease.

## MATERIALS AND METHODS

Detailed methods are provided in the Supplementary materials.

### Cell culture and treatments

HK-2 cells (human renal proximal tubular epithelial cells) (ATCC CRL-2190) were maintained as described previously [30]. Detailed information on the culture and treatment media is provided in the Supplementary materials.

### Statistical analysis

Data are expressed as mean ± standard error of the mean (SEM) of each group. The normality of the distribution and the homogeneity of variance were assessed using the Shapiro–Wilk test and the Levene’s test, respectively. If the normality assumption was met, a comparison between the two groups was conducted using the Student’s *t*-test, with or without Welch correction depending on homogeneity of variance Levene’s test. Otherwise, we applied the Mann–Whitney statistical test. Comparisons between more than two groups were performed using one-way analysis of variance (with Bonferroni’s multiple comparison test) or the Kruskal–Wallis test, depending upon normality of the distribution and homogeneity of variance (with Dunn’s multiple comparisons test). When assessing the interaction between two variables (genotype and treatment), we used linear regression models if normally distributed residuals or quantile regression models for the median expression. Fold-change expression was analysed in logarithmic scale within models. The *P*-values for the pairwise comparisons (or contrasts) of interest were obtained from the regression models.

Statistical analysis was conducted using GraphPad Prism 9 software (GrahPad Software, San Diego, CA, USA) and R Statistical Software [31]. A significance level of .05 was applied.

## RESULTS

### FFAs treatment increases GluN1 expression *in vitro*

**Figure 1:**
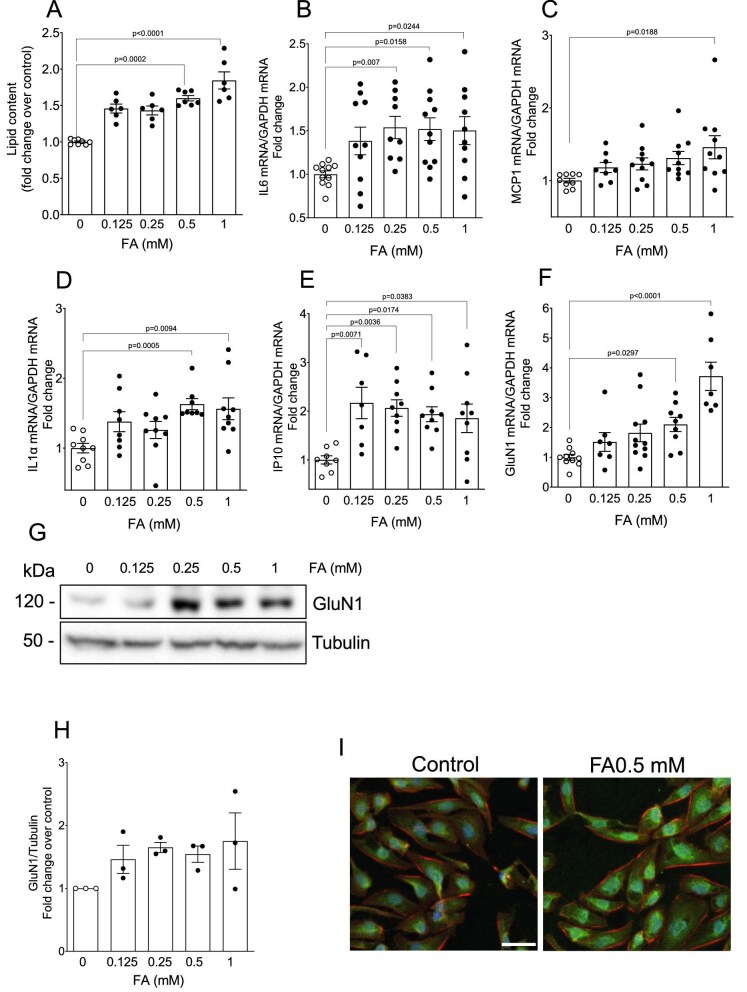
FFAs treatment enhances GluN1 expression in HK-2 cells *in vitro*. HK-2 cells were incubated in serum-free medium or increasing concentrations of FFAs (0.125 mM to 1 mM) (A–H) for 24 h. (**A**) Quantification of cellular lipid content in HK-2 cells after incubation with increasing concentrations of FFAs for 24 h (Oil Red O). Data are presented as mean ± SEM of at least *n* = 3 independent experiments. (**B**–**F**) Total mRNA was extracted from HK-2 cells and mRNA levels were assessed by quantitative real-time PCR. The relative mRNA levels of *GluN1, IL-6, MCP1, IL-1A* and *IP10* were calculated and expressed as fold-change over control after normalizing for GAPDH. Data are presented as mean ± SEM of at least *n* = 3 independent experiments. (**G**) Cell lysates were immunoblotted with antibodies against GluN1. The same samples were reprobed with antibodies against tubulin to ensure equal loading. Representative Western blot of GluN1 subunit (G) and quantitative densitometric analysis (**H**). (**I**) Representative confocal maximum-intensity projection images of HK-2 cells stained for GluN1 (green), F-actin (phalloidin, red) and nuclei (Hoechst, blue), shown as merged channels to visualize overall differences between experimental conditions [control vs FFAs (0.5 mM), 24 h]. Scale bar represents 50 µm.

To evaluate cellular injury and dysfunction upon exposure to free fatty acids (FFAs), we established an *in vitro* model of fat overload in HK-2. FFAs treatment led to a significant increase in lipid accumulation (Fig. 1A) and inflammatory markers expression including interleukin (IL)-6, MCP1, IL-1α and IP10 (Fig. 1B–E). FFA treatment also increased *Kim-1* and *FFAR4* mRNA levels across tested doses (Supplementary Fig. S1). Furthermore, HK-2 exposed to FFAs showed a marked increase of *GluN1* mRNA (Fig. 1F) and protein expression in a dose-dependent manner (Fig. 1G and H). The immunofluorescence showed a visible increase of GluN1 protein in HK-2 cells treated with FFAs, predominantly cytoplasmic/perinuclear (0.5 mM, 24 h; Fig. 1I; Supplementary Fig. S2). These findings suggest a dysregulation of endogenous GluN1 levels following FFA treatment, potentially contributing to fat-induced dysfunction in renal proximal tubular cells.

### Pharmacological inhibition of NMDAR alleviates FFA-induced inflammation and is associated with improved cellular redox homeostasis *in vitro*

**Figure 2:**
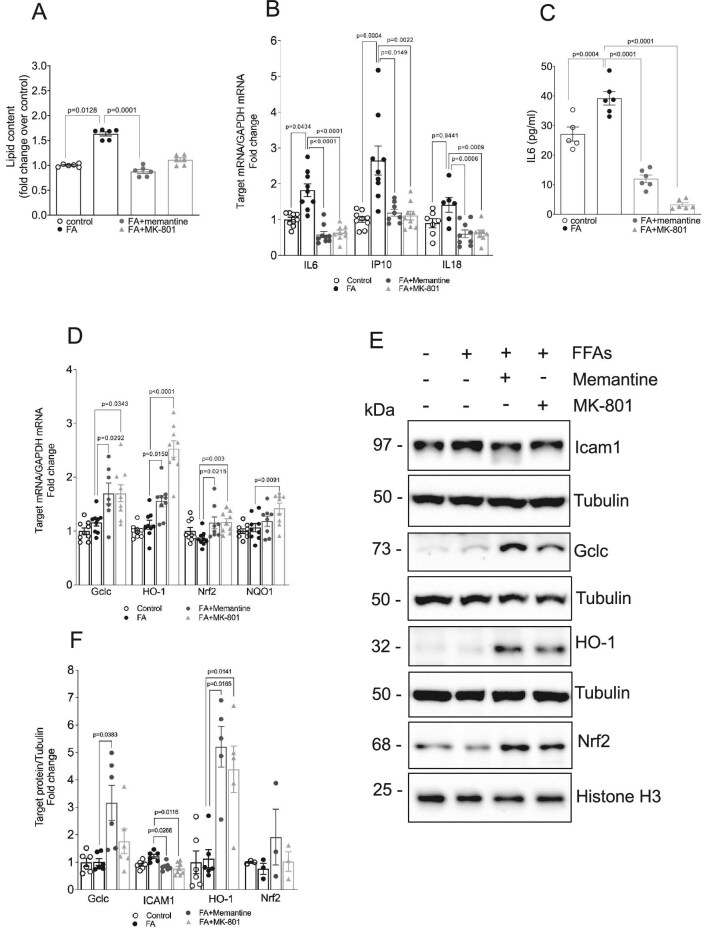
Pharmacological inhibition of NMDAR attenuates FFA-induced inflammation and modulates antioxidant genes *in vitro*. (**A**–**F**) HK-2 cells were incubated with a combined treatment of FFAs (0.5 mM) and NMDAR antagonists (memantine, 0.5 mM or MK-801, 0.5 mM) for 24 h. (A) Quantification of cellular lipid content in HK-2 cells (Oil Red O). Data are presented as mean ± SEM of at least *n* = 3 independent experiments. (B, D) Total mRNA was extracted from HK-2 cells and mRNA levels were assessed by quantitative real-time PCR. The relative mRNA levels of *IL-6, IP10* and *IL-18* (B), *Gclc, HO-1, Nrf2* and *NQO1* (D) were calculated and expressed as fold-change over control after normalizing for GAPDH. Data are presented as mean ± SEM of at least *n* = 3 independent experiments. (C) Measurement of IL-6 secretion into the medium using an enzyme-linked immunosorbent assay. Data were presented as mean ± SEM of at least *n* = 2 independent experiments. (E, F) Cell lysates were immunoblotted with antibodies against Icam1, Gclc, HO-1 and Nrf2. The same samples were reprobed with antibodies against tubulin or Histone 3 to ensure equal loading. (E) Representative Western blots. (F) Quantitative analysis by densitometry. Data are presented as mean ± SEM of at least *n* = 3 independent experiments.

To explore the potential role of NMDAR antagonists in mitigating FFA-induced injury, HK-2 cells were treated with FFAs (0.5 mM) in the presence or absence of memantine or MK-801. Dose optimization showed no cytotoxicity of antagonists alone or in combination with FFAs (Supplementary Fig. S3), while only the 0.5 mM dose efectively reduced lipid accumulation (Fig. 2A; Supplementary Fig. S4).

NMDAR antagonists attenuated the FFA-induced inflammatory response, reducing *IL-6, IP10* and *IL-18* mRNA expression (Fig. 2B), IL-6 secretion (Fig. 2C) and Icam1 protein levels (Fig. 2E and F). Furthermore, treatment with NMDAR antagonists was associated with enhanced expression of the transcription factor Nrf-2 and its downstream antioxidant defense genes *HO-1, Gclc* and *NQO-1* at both the mRNA (Fig. 2D) and protein levels (Fig. 2E and F).

These results suggest that pharmacological inhibition of NMDAR attenuates FFA-induced lipid accumulation and inflammation, concomitant with increased expression of Nrf-2-related antioxidant pathways.

### Overexpression of GluN1 promotes inflammation and impairs antioxidant defense *in vitro*

**Figure 3:**
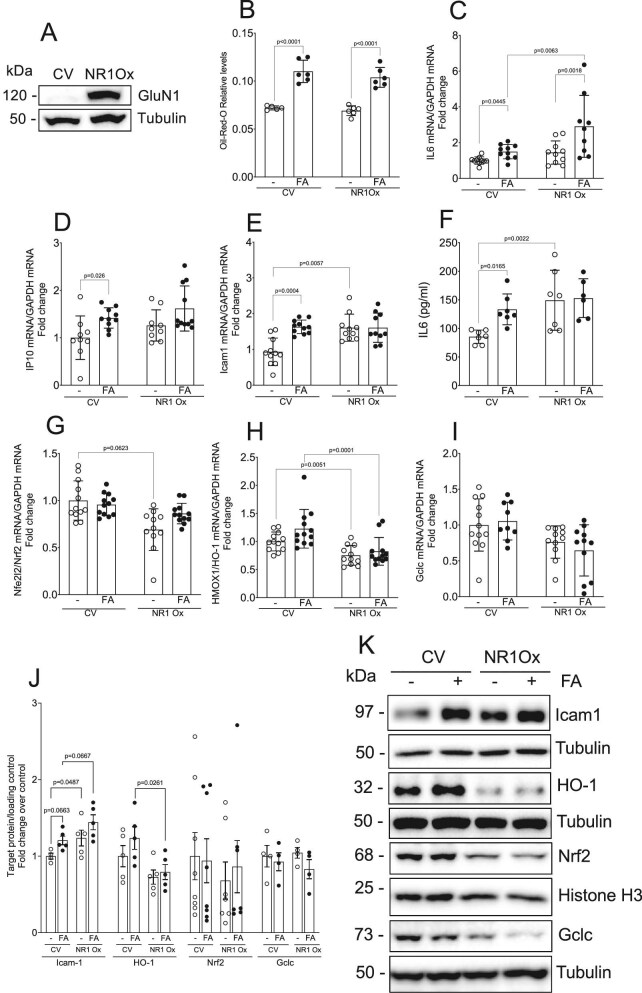
*In vitro* overexpression of GluN1 triggers enhanced inflammatory response and reduces antioxidant defense. (**A**–**K**) GluN1 was stably overexpressed in HK-2 cells, and the cells were incubated with FFAs (0.5 mM) or left untreated for 24 h. (B) Quantification of cellular lipid content in HK-2 cells (Oil Red O). Data are presented as mean ± SEM of at least *n* = 3 independent experiments. (C–E, G–I) Total mRNA was extracted from HK-2 cells and mRNA levels were assessed by quantitative real-time PCR. The relative mRNA levels of *IL-6, IP10, Icam1, Gclc, Nrf2* and *HO-1* were calculated and expressed as fold-change over control after normalizing for GAPDH. Data are presented as mean ± SEM of at least *n* = 3 independent experiments. (F) Measurement of IL-6 secretion into the medium using an enzyme-linked immunosorbent assay. Data were presented as mean ± SEM of at least *n* = 3 independent experiments. (J, K) Cell lysates were immunoblotted with antibodies against Icam1, HO-1, Nrf2 and Gclc. The same samples were reprobed with antibodies against tubulin or Histone 3 to ensure equal loading. (J) Representative Western blots. (K) Quantitative analysis by densitometry. Data are presented as mean ± SEM of at least *n* = 3 independent experiments. NR1Ox, cells overexpressing GluN1 subunit.

Next, we stably overexpressed GluN1 in HK-2 cells (Fig. 3A), after which the cells were incubated with FFAs or were left untreated for 24 h. Overexpression of GluN1 did not affect the accumulation of intracellular lipids (Fig. 3B), however it did impact the cell´s inflammatory state (Fig. 3C–F, J and K). NR1Ox cells exhibited significantly higher IL-6 secretion (Fig. 3F) and an increase of Icam1 (Fig. 3J and K) compared with control vector (CV). Although FFAs led to a higher expression of inflammatory markers (Fig. 3C–F, J and K) in cells infected with CV, this effect was not observed in NR1Ox cells, for which the levels of *IP10* and *Icam1* mRNA expression (Fig. 3D, E, J and K), and IL-6 secretion (Fig. 3F) were not further elevated by FFAs treatment and were comparable to those of the CV + FFAs condition. However, *IL-6* mRNA levels were significantly higher in the NR1Ox + FFAs than in the CV + FFAs group (Fig. 3C).

GluN1 overexpression reduced *Nrf2* and *HO-1* mRNA (Fig. 3G and H) and protein levels (Fig. 3J and K), with a trend toward decreased *Gclc* mRNA levels in NR1Ox cells (Fig. 3I). Moreover, GluN1 overexpression increased susceptibility to FFA-induced loss of antioxidant protection, significantly reducing *HO-1* mRNA (Fig. 3H) and protein levels (Fig. 3J and K). These results suggest that overexpression of GluN1 amplifies the inflammatory response and impairs antioxidant defense mechanisms *in vitro*. Consistent with a complex regulatory role of GluN1/NMDAR in this setting, GluN1 knockdown in HK-2 cells was associated with increased inflammatory and reduced antioxidant markers (Supplementary Fig. S5).

### NMDAR antagonists mitigate p38 activation triggered by FFAs *in vitro*

**Figure 4:**
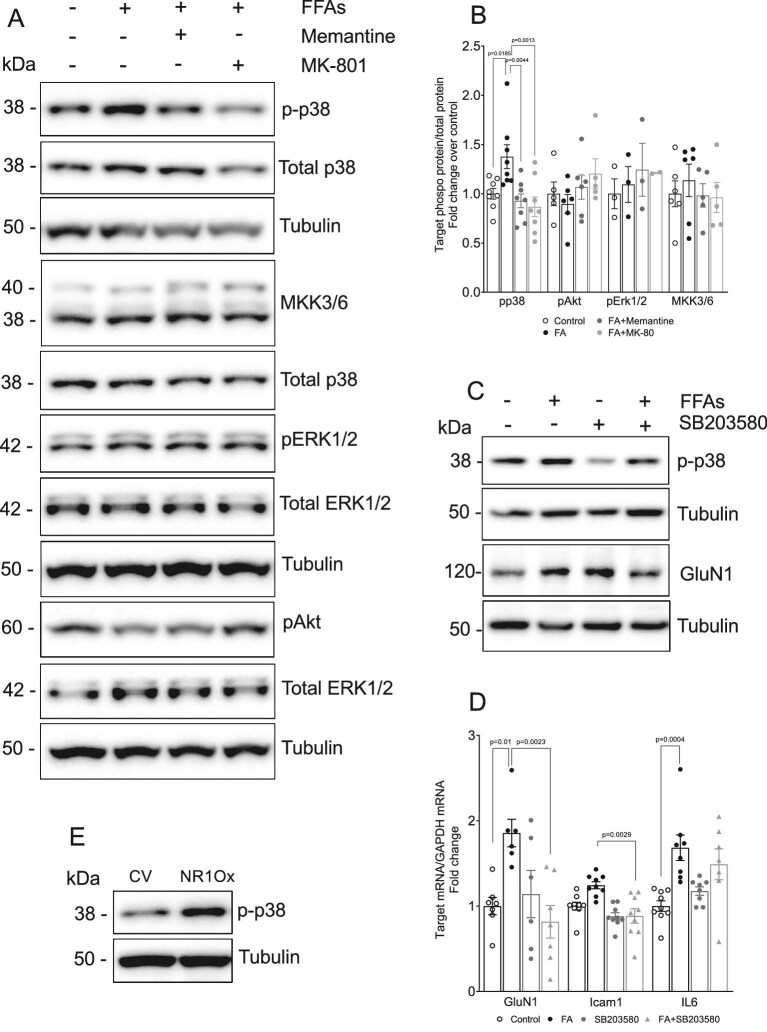
NMDAR antagonists suppress FFA-induced p38 activation *in vitro.* (**A, B**) HK-2 cells were incubated with a combined treatment of FFAs (0.5 mM) and NMDAR antagonists (memantine, 0.5 mM or MK-801, 0.5 mM) for 60 min. Cell lysates were immunoblotted with antibodies against p-p38, pErk1/2, pAkt, pMKK3/6, total ERK1/2, total p38 and tubulin. (A) Representative Western blots. (B) Quantitative analysis by densitometry. Data were normalized to total Erk1/2 or total p38 and presented as mean ± SEM of *n* = 3 independent experiments. (**C, D**) HK-2 cells were pretreated for 1 h with SB203580 (20 µM/L), after which cells were incubated either with serum-free medium or FFAs (0.5 mM) for 24 h. (C) Representative Western blots showing levels of p-p38 and GluN1 in HK-2 cells. (D) Total mRNA was extracted from HK-2 cells and mRNA levels were assessed by quantitative real-time PCR. The relative mRNA levels of *GluN1, Icam1* and *IL-6* were calculated and expressed as fold-change over control after normalizing for GAPDH. Data are presented as mean ± SEM of at least *n* = 3 independent experiments. (E) Representative Western blot showing levels of p-p38 in HK-2 cells overexpressing GluN1 subunit.

To explore the mechanism underlying the effect of NMDAR antagonists on FFA-induced cell dysfunction, we assessed the MAPK and PI3K-Akt activities, which are involved in various nephropathies and in lipid metabolism processes [8, 32]. FFA treatment did not alter pErk1/2 and pAkt levels but significantly increased p-p38 in HK-2 cells, an effect that was reduced by co-treatment with NMDAR antagonists (Fig. 4A and B). Phosphorylation of p38 MAPK kinases, pMKK3 and pMKK6, remained unchanged by FFAs or antagonist treatment (Fig. 4A and B). Overexpression of GluN1 under basal conditions altered p38 activity (Fig. 4E), similar to FFA treatment, despite no increase in lipid accumulation (Fig. 3B).

Next, we wished to investigate the mechanism that stands behind the FFA-induced increase in GluN1 expression. Inhibition of p38 with SB203580 inhibitor attenuated FFA-induced increase in GluN1 (Fig. 4C and D) and reduced Icam1 expression (Fig. 4D), with a trend toward lower IL-6 (Fig. 4D), indicating that the MAPK–p38 pathway mediates FFA-induced inflammatory response in HK-2 cells.

### Renal GluN1 expression increases in CKD patients

**Figure 5:**
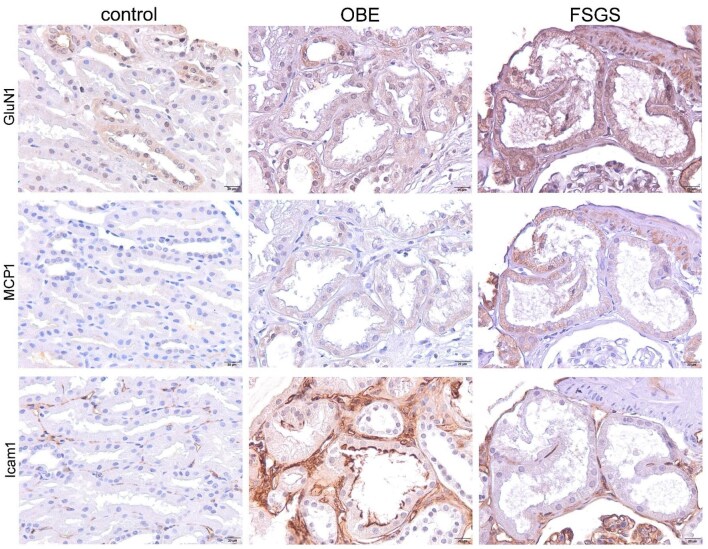
Increased renal GluN1 expression in CKD patients. Representative photomicrographs of immunoperoxidase staining for GluN1, MCP1 and Icam1 in kidney sections of patients with obesity-related glomerulopathy with proteinuria (OBE) and patients with FSGS versus kidney sections with no evidence of pathology. Scale bar represents 20 µm.

To investigate the involvement of NMDAR in the development of CKD in human patients, we assessed the protein levels of GluN1 in kidney biopsies of obese patients with obesity-related glomerulopathy with proteinuria and focal segmental glomerulosclerosis (FSGS) patients with hyperlipidemia (Supplementary Table S3). As controls, we used renal biopsies confirmed to have normal histology upon examination. Interestingly, GluN1 expression was found to be upregulated in the kidneys of both patient groups—obese patients with proteinuria and FSGS patients with hyperlipidemia—suggesting a potential link between lipid-driven kidney injury and GluN1 expression (Fig. 5). Furthermore, we found elevated protein levels of inflammatory markers MCP1 and Icam1 in tubular areas of both disease conditions (Fig. 5; Supplementary Table S4).

### Renal GluN1 expression increases in OIKD mouse model

**Figure 6:**
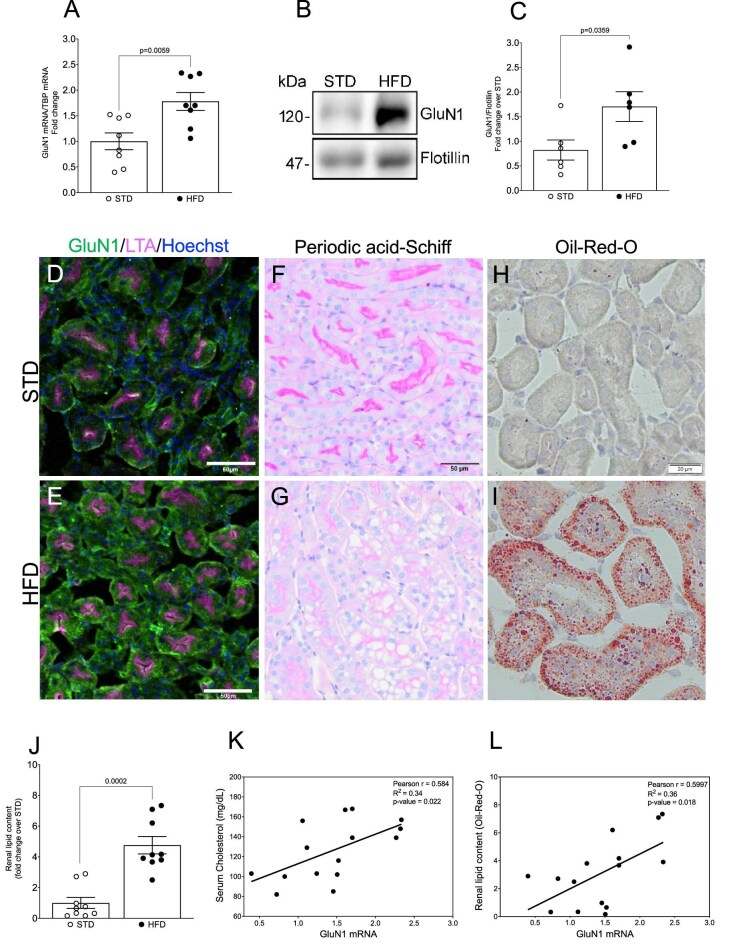
Renal expression of GluN1 increases in OIKD mouse model. (**A**) Total mRNA was extracted from the kidneys of mice fed an STD or an HFD, and mRNA levels of *GluN1* were determined by quantitative real-time PCR. Relative mRNA levels were calculated and expressed as fold-change over STD (value = 1.0) after normalizing for TBP. Data are presented as mean ± SEM of 7–9 mice/group. (**B, C**) Representative immunoblots showing renal *GluN1* mRNA expression (B) and quantitative densitometric analysis (C). Data are presented as mean ± SEM of 6 mice/group. (**D, E**) Immunofluorescence staining for GluN1 in mouse kidney after HFD feeding. Representative images of kidney sections from mice fed an STD (D) or an HFD (E) stained for GluN1 (green) with lotus tetragonolobus agglutinin (LTA, magenta) and counterstained for Hoechst to visualize nuclei. LTA is a marker of RPTECs and no difference in its expression was observed between STD and HFD-fed mice. Scale bar 50 µm. (**F**–**I**) Representative photomicrographs of periodic acid–Schiff staining (PAS) (F, G) and Oil Red O staining (H, I) of paraffin-embedded (F, G) and frozen (H, I) kidney sections from mice fed an STD (F, H) or an HFD diet (G, I). Scale bar represents 50 µm (F, G) or 20 µm (H, I). (**J**) Quantification of renal lipid content (Oil Red O) in the mouse kidneys. Data are presented as mean ± SEM of 9 mice/group (fold-change over STD). (**K, L**) Scatter plots show correlation analysis between renal *GluN1* mRNA levels and serum cholesterol (K) and/or renal lipid content (L). Serum cholesterol and renal lipid content were assessed by standard methods explained in the Materials and methods sections. The Spearman correlation coefficient (R) and *P*-value are shown. STD, standard diet-fed mice.

To analyze the expression levels of GluN1 in OIKD in mice, C57BL/6J mice were maintained on a high-fat diet (HFD) for 10 weeks. HFD led to a prominent increase in renal *GluN1* mRNA (Fig. 6A) and protein expression (Fig. 6B and C), confirmed by immunofluorescence staining and localized predominantly in LTA-positive proximal tubules (Fig. 6D and E). Importantly, HFD led to a marked accumulation of neutral lipids in renal tubules of HFD-fed mice compared with controls (Fig. 6F–J). The correlation analysis showed a significant association between renal *GluN1* mRNA expression and total serum cholesterol levels (R^2^ = 0.34; R = 0.584; *P* = .02) (Fig. 6K), as well as the renal lipid content (R^2^ = 0.36; R = 0.5997; *P* = .018) (Fig. 6L).

### NMDAR antagonists do not affect systemic lipid levels in OIKD mouse model

**Figure 7:**
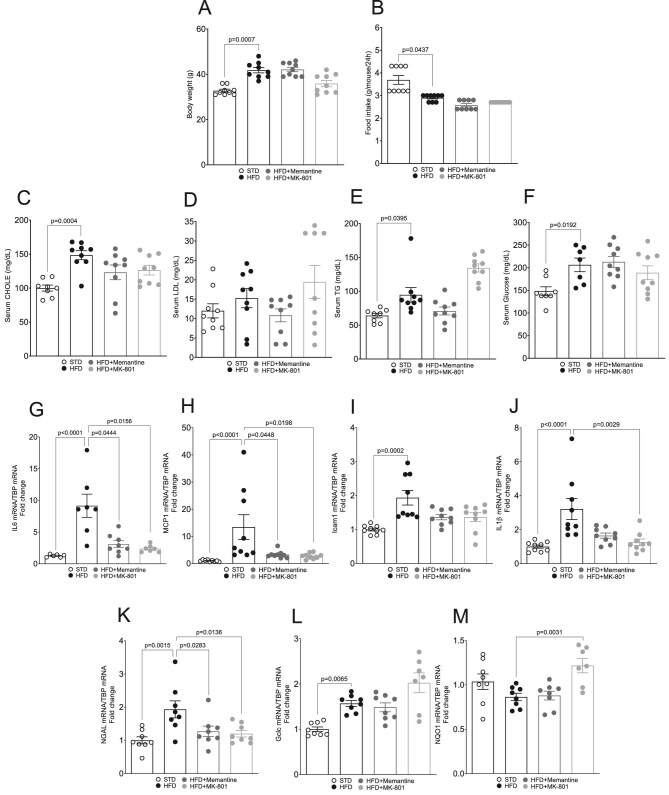
NMDAR antagonists modulate kidney inflammation and antioxidant genes in HFD-fed mice. (**A**–**M**) C57BL/6J mice were fed an STD, HFD or HFD + memantine and HFD + MK-801, and were sacrificed after 10 weeks of feeding. (A–F) Body weight was measured every week throughout the whole experiment (A). Individual food intake was measured at three time points during the experiment (**B**). Serum cholesterol (C), LDL (D), triglyceride (E) and glucose (F) were measured by standard methods explained in the Materials and methods section. Data are presented as mean ± SEM of 8–9 mice/group. (G–M) Total mRNA was extracted from the kidneys and mRNA levels were assessed by quantitative real-time PCR. The relative mRNA levels of *IL-6, MCP1, Icam1, IL-1β, NGAL, Gclc* and *NQO1* were calculated and expressed as fold-change over STD (value = 1.0) after normalizing for TBP. Data are presented as mean ± SEM of 7–9 mice/group. STD, standard diet-fed mice.

To explore whether the renoprotective effect of NMDAR inhibition *in vitro* could have a role in an *in vivo* model of OIKD in mice, C57BL/6J animals were fed an HFD for 10 weeks and treated with NMDAR antagonists. NMDAR antagonists did not significantly affect body weight (Fig. 7A) or food intake (Fig. 7B), except for MK-801, which caused a modest reduction in body weight (Fig. 7A). HFD-fed mice developed pronounced hypercholesterolemia (Fig. 7C–E) and elevated fasting glucose (Fig. 7F), while the administration of NMDAR antagonists did not have any effect on lipid profile or glucose levels (Fig. 7C–F). The observed dyslipidemia in obese mice correlated with increased body weight (Fig. 7A), despite a slight reduction in food intake (Fig. 7B).

### NMDAR antagonists alleviate HFD-induced renal inflammation

**Figure 8:**
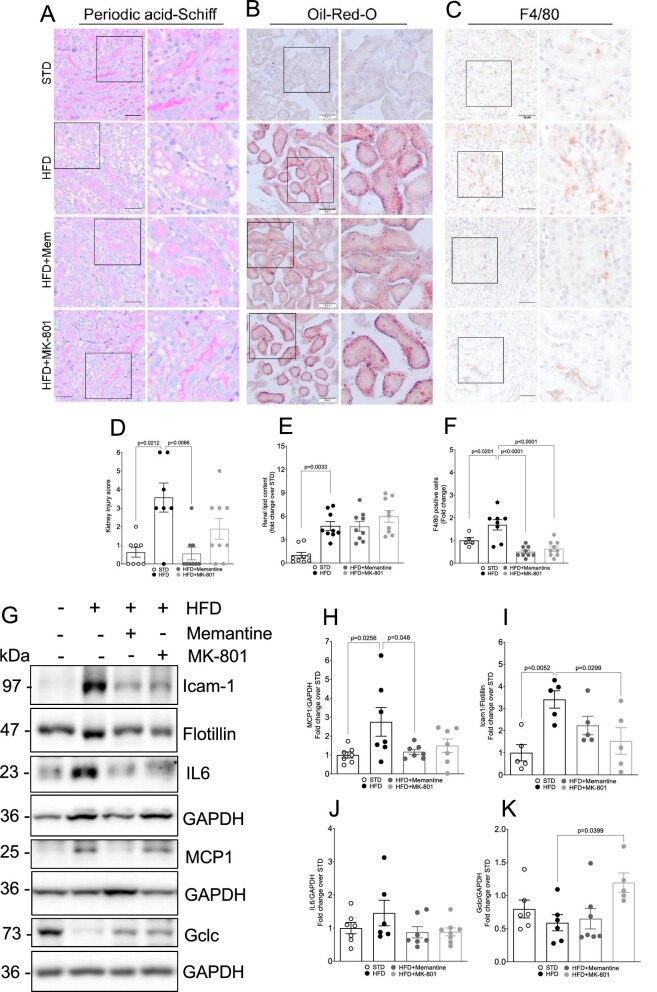
NMDAR antagonists reduce renal injury and macrophage infiltration in the kidneys of HFD-fed mice. (**A**–**K**) C57BL/6J mice were fed an STD, HFD or HFD + memantine and HFD + MK-801, and were sacrificed after 10 weeks of feeding. (A–C) Representative photomicrographs of periodic acid–Schiff staining (PAS) (A), Oil Red O staining (B) and immunohistochemistry for F4/80 (C) in mouse kidneys. Scale bar represents 50 µm (A–C). (D) Kidney injury score was assessed as explained in the Materials and methods section. (E, F) Quantification of renal lipid content (Oil Red O) (E) and F4/80 staining (F) in the mouse kidneys. Data are presented as mean ± SEM of 7–9 mice/group (fold-change over STD). STD, standard diet-fed mice. (G) Kidney lysates were immunoblotted with antibodies against Icam1, IL-6, MCP1 and Gclc. The same samples were reprobed with antibodies against Flotillin and GAPDH to ensure equal loading. (G) Representative Western blot analysis of Icam1, IL-6, MCP1 and Gclc from kidney extracts. (H–K) Quantitative analysis by densitometry.

In vivo, HFD significantly increased renal *IL-6, MCP1, Icam1* and *IL-1β* mRNA expression (Fig. 7G–J), which was effectively reduced by the co-treatment with NMDAR antagonists (Fig. 7G–J). Similar effects were observed at the protein level, where HFD caused elevated levels of inflammatory markers in the kidney (Fig. 8A–E), while antagonists managed to alleviate these changes (Fig. 8A–E). In our mouse model, HFD induced significant infiltration of macrophages in the renal tissue, seen as a positive immunoreactivity for F4/80 (Fig. 8C and F). Treatment with either memantine or MK-801 abolished HFD-induced macrophage accumulation in the interstitium (Fig. 8C and F), suggesting that both NMDAR antagonists were effective in alleviating obesity-induced renal inflammation.

### NMDAR antagonists ameliorate kidney injury and modulate antioxidant gene expression

HFD feeding significantly increased renal *NGAL* mRNA expression (Fig. 7K), which was reduced by NMDAR antagonists (Fig. 7K). Ten weeks of HFD feeding caused an evident renal parenchyma damage, characterized by increased cytoplasmic vacuolation and the loss of brush border in renal proximal tubular cells (Fig. 8A and D), as well as neutral lipid accumulation in the kidney (Fig. 8B and E). NMDAR antagonists, particularly memantine, mitigated kidney damage (Fig. 8A and D), but failed to reduce lipid accumulation in the kidney (Fig. 8B and E). MK-801 induced a clear upregulation of renal NQO-1 and Gclc mRNA (Fig. 7L and M), while memantine did not produce the same effect. This was further corroborated at the protein level, where MK-801-treated animals demonstrated elevated levels of Gclc protein in the kidney (Fig. 8G and K).

## DISCUSSION

In this study, we describe a novel role for NMDAR in OIKD and demonstrate that two structurally different NMDAR antagonists protect against obesity-associated inflammation and redox imbalance.

Obesity is a major driver of kidney injury, mainly due to its association with increased chronic inflammation and oxidative stress [7, 11]. *In vitro*, FFA exposure led to lipid accumulation, inflammation and upregulation of GluN1, implicating this subunit in tubular epithelial cells dysfunction. Consistently, renal GluN1 expression was elevated in a mouse model of OIKD and positively correlated with serum cholesterol and renal lipid content. GluN1 levels were also elevated in kidney biopsies from obese patients with proteinuria and from FSGS patients with hyperlipidemia. We included FSGS because it represents a common histopathological outcome of obesity-related kidney injury [33] and has been used in recent studies on obesity-induced CKD to validate tubular receptor upregulation *in situ* [34], highlighting the translational relevance of our findings. Although glomerular injury is a key characteristic of obesity-related kidney disease, these studies, like ours, focused on proximal tubular cells due to their high susceptibility to lipid-induced stress and their important role in mediating inflammatory and oxidative responses. Alongside earlier reports showing that lipids modulate NMDAR expression in the brain [28, 35], our data support the mechanistic link between lipid accumulation and GluN1 upregulation in OIKD.

To explore the potential role of NMDAR in kidney epithelial injury, we overexpressed GluN1 *in vitro* and challenged the cells with FFAs. GluN1 overexpression alone triggered inflammation and impaired antioxidant defense, while FFAs treatment did not further aggravate these effects, pointing to a prevailing effect of GluN1 in modulating these effects. Previous studies have shown that overexpression of NMDAR induced inflammation and oxidative stress in diverse models [36–40]. Jeon *et al*. demonstrated that GluN1 overexpression in macrophages promoted M1 polarization [36], while forebrain-targeted GluN2 overexpression led to tissue injury and inflammation in transgenic mice [37]. Our findings are in line with these reports, demonstrating that GluN1 contributes to inflammation and redox homeostasis disturbance in kidney epithelial cells, highlighting NMDAR as a pathogenic driver and potential therapeutic target in OIKD. Reports of protective NMDAR signaling have been described in certain experimental settings, which are mechanistically distinct from the model examined in the present study.

Having established GluN1’s pro-inflammatory role and its association with reduced antioxidant gene expression in HK-2 cells, we next examined the therapeutic potential of two structurally different NMDAR antagonists, memantine and MK-801. NMDAR antagonists decreased FFA-induced lipid accumulation and inflammation *in vitro*. Additionally, antagonists upregulated Nrf-2 and its downstream antioxidant genes, highlighting their role in enhancing cellular defense mechanisms against lipid-induced disturbance of redox homeostasis. The renoprotective effect of NMDAR inhibition was corroborated in a mouse model of OIKD. Memantine and MK-801 effectively reduced renal inflammation by lowering IL-6, MCP1, Icam1 and IL-1β expression and by inhibiting macrophage infiltration. Both antagonists also attenuated NGAL expression, while MK-801 further enhanced NQO1-1 and Gclc, supporting its role in antioxidant defense in OIKD. Unlike *in vitro* findings, NMDAR antagonists did not reduce systemic or renal lipid accumulation induced by HFD feeding, suggesting they act by modulating inflammation and oxidative stress rather than directly modulating systemic lipid levels. Our findings indicate that NMDAR antagonism may protect the kidney in lipid-associated conditions independent of effects on lipid metabolism. Additionally, NMDAR antagonists did not have any effect on HFD-induced increase of body weight, suggesting that their protective effect was not driven through changes in overall weight. Although MK-801-treated mice showed slightly higher serum and low-density lipoprotein levels, these differences were not statistically significant and their mechanistic basis remains unclear.

Multiple studies have demonstrated implication of the MAPK and PI3K/Akt pathways in the pathogenesis of various nephropathies [30, 41–43], as well as their involvement in processes related to lipid metabolism [32, 44]. Furthermore, FFAs, both saturated and unsaturated, have been shown to activate MAPK-p38 pathway [45–47], a key signaling molecule involved in inflammation and stress responses [48]. In the present study, FFA treatment led to a significant increase of p-p38 in HK-2 cells, while the levels of pErk1/2 and pAkt stayed unchanged. Despite elevated phosphorylation of p38 induced by FFAs, levels of pMKK3/6 stayed stable. Co-treatment with NMDAR antagonists reduced p38 phosphorylation, while they did not have an effect on pMKK3/6 levels. Importantly, overexpression of GluN1 alone in HK-2 cells affected the activity of p38 at basal state, indicating that GluN1 may act upstream of p38 in response to FFAs. These findings demonstrate the role of NMDAR in regulating MAPK–p38 pathway in kidney cells and are consistent with previous research conducted in both the heart and brain [49–51]. Namely, Liu *et al*. [49] demonstrated that the NMDAR signaling enhanced p38 MAPK phosphorylation, which was abolished by the treatment with MK-801 in cardiomyocytes. Interestingly, Ahn *et al*. [51] confirmed that NMDAR exerts a more potent regulatory effect on the p38 pathway than on the Erk1/2 and MEK1 pathways in the rat hippocampus. Furthermore, it has been shown that GluN2B-containing NMDAR was responsible for p38 MAPK activation and neurotoxicity in mature neurons [50]. The present study reports the role for GluN1 as a non-canonical mediator of p38 MAPK activation in response to FFA in HK-2 cells, working independently of MKK3/6. In addition, FFA increased *HAVCR1* and *FFAR4* mRNA levels, consistent with FFA-response signaling, although an upstream role was not assessed. To investigate the mechanisms underlying FFA-induced increase in GluN1 expression, we treated cells with the p38 kinase inhibitor, SB203580. We found that SB203580 markedly attenuated GluN1 expression caused by FFA treatment, alongside the FFA-induced inflammatory response, implying a feed-forward loop whereby p38 activity increases GluN1 expression, further boosting inflammatory cascade in kidney cells.

A limitation of our study is that we did not employ a genetic knockout model of NMDAR. However, given that shRNA-mediated downregulation of GluN1 in HK-2 cells exacerbated inflammation and oxidative stress, genetic deletion may not reliably reflect the therapeutic potential of receptor inhibition. We thus focused on pharmacological antagonism *in vitro* and *in vivo*, and complementary gain- and loss-of-function approaches *in vitro*, which together support the role of NMDAR signaling in OIKD. Furthermore, future work should define whether obesity can change NMDAR co-agonist availability and/or tubular membrane potential to promote receptor activation.

Another key limitation is the use of systemic antagonists, which target NMDARs in the kidney as well as in peripheral tissues and the CNS. Keeping in mind that both NMDAR antagonists cross the blood–brain barrier, it remains unclear precisely where they exert their beneficial effects in reducing renal complications in OIKD mice. Nevertheless, our findings highlight the potential of using non-harmful and clinically approved drugs that can inhibit NMDAR receptors, such as memantine, and provide a practical advantage over unproven treatment strategies.

In summary, our study identifies a novel function for GluN1 in OIKD and demonstrates that NMDAR antagonism effectively reduces lipid-induced inflammation, restores antioxidant defense mechanisms and mitigates renal injury, both *in vitro* and *in vivo*. Pharmacological inhibition of NMDARs may represent a therapeutic strategy that protects the kidney from injury driven by obesity and lipid overload, providing a foundation for future translational studies.

## Supplementary Material

gfag027_Supplemental_File

## Data Availability

The authors declare that the main data supporting the findings of this study are included within the manuscript or supplementary information, or are available from the corresponding author upon reasonable request.

## References

[1] World Health Organization. WHO acceleration plan to stop obesity. Geneva: World Health Organization; 2023. ISBN: 978-92-4-007563-4. Available at: https://www.who.int/publications/i/item/9789240075634 [1 March 2026; date last accessed].

[2] Ejerblad E, Fored CM, Lindblad P et al. Obesity and risk for chronic renal failure. J Am Soc Nephrol 2006;17:1695–702. 10.1681/ASN.2005060638 16641153

[3] Sharma I, Liao Y, Zheng X et al. New pandemic: obesity and associated nephropathy. Front Med (Lausanne) 2021;8:673556. 10.3389/fmed.2021.673556 34268323 PMC8275856

[4] Nguyen A, Khafagy R, Gao Y et al. Association between obesity and chronic kidney disease: multivariable mendelian randomization analysis and observational data from a bariatric surgery cohort. Diabetes 2023;72:496–510. 10.2337/db22-0696 36657976 PMC10197093

[5] VD D’A, Chagnac A, de Vries AP et al. Obesity-related glomerulopathy: clinical and pathologic characteristics and pathogenesis. Nat Rev Nephrol 2016;12:453–71. 27263398 10.1038/nrneph.2016.75

[6] Caus M, Eritja À, Bozic M. Role of microRNAs in obesity-related kidney disease. Int J Mol Sci 2021;22:11416. 10.3390/ijms222111416 PMC8583993 34768854

[7] Udi S, Hinden L, Earley B et al. Proximal tubular cannabinoid-1 receptor regulates obesity-induced CKD. J Am Soc Nephrol 2017;28:3518–32. 10.1681/ASN.2016101085 28860163 PMC5698062

[8] Eritja À, Caus M, Belmonte T et al. microRNA expression profile in obesity-induced kidney disease driven by high-fat diet in mice. Nutrients 2024;16:691. 10.3390/nu16050691 PMC10934936 38474819

[9] Chien MJ, Li SJ, Wong SC et al. Determination of mitochondrial functions and damage in kidney in female LeeSung minipigs with a high-fat diet-induced obesity. Arch Physiol Biochem 2023;129:1289–97. 10.1080/13813455.2021.1949022 34338085

[10] Moorhead JF, Chan MK, El-Nahas M et al. Lipid nephrotoxicity in chronic progressive glomerular and tubulo-interstitial disease. Lancet 1982;2:1309–11. 10.1016/S0140-6736(82)91513-6 6128601

[11] Stemmer K, Perez-Tilve D, Ananthakrishnan G et al. High-fat-diet-induced obesity causes an inflammatory and tumor-promoting microenvironment in the rat kidney. Dis Model Mech 2012;5:627–35. 22422828 10.1242/dmm.009407 PMC3424460

[12] Kim I, Kim HR, Kim JH et al. Beneficial effects of Allium sativum L. stem extract on lipid metabolism and antioxidant status in obese mice fed a high-fat diet. J Sci Food Agric 2013;93:2749–57. 10.1002/jsfa.6094 23606129

[13] Deji N, Kume S, Araki S et al. Structural and functional changes in the kidneys of high-fat diet-induced obese mice. Am J Physiol Renal Physiol 2009;296:F118–26. 10.1152/ajprenal.00110.2008 18971213

[14] Haddad JJ. N-methyl-D-aspartate (NMDA) and the regulation of mitogen-activated protein kinase (MAPK) signaling pathways: a revolving neurochemical axis for therapeutic intervention? Prog Neurobiol 2005;77:252–82. 10.1016/j.pneurobio.2005.10.008 16343729

[15] Bellone C, Nicoll RA. Rapid bidirectional switching of synaptic NMDA receptors. Neuron 2007;55:779–85. 10.1016/j.neuron.2007.07.035 17785184

[16] Traynelis SF, Wollmuth LP, McBain CJ et al. Glutamate receptor ion channels: structure, regulation, and function. Pharmacol Rev 2010;62:405–96. 10.1124/pr.109.002451 20716669 PMC2964903

[17] Rebola N, Srikumar BN, Mulle C. Activity-dependent synaptic plasticity of NMDA receptors. J Physiol 2010;588:93–9. 10.1113/jphysiol.2009.179382 19822542 PMC2821550

[18] Luo J, Wang Y, Yasuda RP et al. The majority of N-methyl-D-aspartate receptor complexes in adult rat cerebral cortex contain at least three different subunits (NR1/NR2A/NR2B). Mol Pharmacol 1997;51:79–86. 10.1124/mol.51.1.79 9016349

[19] Beaurain M, Salabert AS, Payoux P et al. NMDA receptors: distribution, role, and insights into neuropsychiatric disorders. Pharmaceuticals (Basel) 2024; 17:1265. 10.3390/ph17101265 PMC11509972 39458906

[20] Valdivielso JM, Eritja À, Caus M et al. Glutamate-gated NMDA receptors: insights into the function and signaling in the kidney. Biomolecules 2020;10:1051. 10.3390/biom10071051 PMC7407907 32679780

[21] Bozic M, Valdivielso JM. Calcium signaling in renal tubular cells. Adv Exp Med Biol 2012;740:933–44. 22453977 10.1007/978-94-007-2888-2_42

[22] Bozic M, de Rooij J, Parisi E et al. Glutamatergic signaling maintains the epithelial phenotype of proximal tubular cells. J Am Soc Nephrol 2011;22:1099–111. 10.1681/ASN.2010070701 21597037 PMC3103729

[23] Deng A, Valdivielso JM, Munger KA et al. Vasodilatory N-methyl-D-aspartate receptors are constitutively expressed in rat kidney. J Am Soc Nephrol 2002;13:1381–4. 10.1097/01.ASN.0000013293.11876.4E 11961027

[24] Bądzyńska B, Zakrocka I, Sadowski J et al. Effects of systemic administration of kynurenic acid and glycine on renal haemodynamics and excretion in normotensive and spontaneously hypertensive rats. Eur J Pharmacol 2014;743:37–41. 25263305 10.1016/j.ejphar.2014.09.020

[25] Roshanravan H, Kim EY, Dryer SE. NMDA receptors as potential therapeutic targets in diabetic nephropathy: increased renal NMDA receptor subunit expression in Akita mice and reduced nephropathy following sustained treatment with memantine or MK-801. Diabetes 2016;65:3139–50. 10.2337/db16-0209 27388219 PMC5033270

[26] Singh AP, Singh N, Bedi PMS. Estradiol mitigates ischemia reperfusion-induced acute renal failure through NMDA receptor antagonism in rats. Mol Cell Biochem 2017;434:33–40. 10.1007/s11010-017-3034-9 28432550

[27] Parisi E, Bozic M, Ibarz M et al. Sustained activation of renal N-methyl-D-aspartate receptors decreases vitamin D synthesis: a possible role for glutamate on the onset of secondary HPT. Am J Physiol Endocrinol Metab 2010;299:E825–31. 10.1152/ajpendo.00428.2010 20823451 PMC2980358

[28] Paul SM, Doherty JJ, Robichaud AJ et al. The major brain cholesterol metabolite 24(S)-hydroxycholesterol is a potent allosteric modulator of N-methyl-D-aspartate receptors. J Neurosci 2013;33:17290–300. 10.1523/JNEUROSCI.2619-13.2013 24174662 PMC3812502

[29] Song T, Zhao J, Ma X et al. Role of sigma 1 receptor in high fat diet-induced peripheral neuropathy. Biol Chem 2017;398:1141–9. 10.1515/hsz-2017-0117 28525360

[30] Bozic M, Caus M, Rodrigues-Diez RR et al. Protective role of renal proximal tubular alpha-synuclein in the pathogenesis of kidney fibrosis. Nat Commun 2020;11:1943. 10.1038/s41467-020-15732-9 32327648 PMC7181766

[31] R Development Core Team . R: A Language and Environment for Statistical Computing. R Foundation for Statistical Computing, Vienna, Austria, 2024. Available from: https://www.R-project.org/ [1 March 2026; date last accessed].

[32] Wen X, Zhang B, Wu B et al. Signaling pathways in obesity: mechanisms and therapeutic interventions. Signal Transduct Target Ther 2022;7:298. 10.1038/s41392-022-01149-x 36031641 PMC9420733

[33] Hao M, Lv Y, Liu S et al. The new challenge of obesity—obesity-associated nephropathy. Diabetes Metab Syndr Obes 2024;17:1957–71. 10.2147/DMSO.S433649 38737387 PMC11086398

[34] Udi S, Hinden L, Ahmad M et al. Dual inhibition of cannabinoid CB. Br J Pharmacol 2020;177:110–27. 10.1111/bph.14849 31454063 PMC6976880

[35] Mateos L, Akterin S, Gil-Bea FJ et al. Activity-regulated cytoskeleton-associated protein in rodent brain is down-regulated by high fat diet in vivo and by 27-hydroxycholesterol in vitro. Brain Pathol 2009;19:69–80. 10.1111/j.1750-3639.2008.00174.x 18503570 PMC8095555

[36] Jeon HJ, Byun JK, Lee SB et al. N-methyl-D-aspartate receptors induce M1 polarization of macrophages: feasibility of targeted imaging in inflammatory response in vivo. Cell Biosci 2023;13:69. 10.1186/s13578-023-01007-5 36998073 PMC10064586

[37] Wei F, Wang GD, Kerchner GA et al. Genetic enhancement of inflammatory pain by forebrain NR2B overexpression. Nat Neurosci 2001;4:164–9. 10.1038/83993 11175877

[38] Li Q, Qin M, Tan Q et al. MicroRNA-129-1-3p protects cardiomyocytes from pirarubicin-induced apoptosis by down-regulating the GRIN2D-mediated Ca. J Cell Mol Med 2020;24:2260–71. 10.1111/jcmm.14908 31957170 PMC7011137

[39] Thongsepee N, Himakhun W, Kankul K et al. Monosodium glutamate altered renal architecture and modulated expression of NMDA-R, eNOS, and nNOS in normotensive and hypertensive rats. Food Chem Toxicol 2024;189:114763. 10.1016/j.fct.2024.114763 38797315

[40] Hussain S, Bahadar H, Khan MI et al. Modulation of oxidative stress/NMDA/nitric oxide pathway by topiramate attenuates morphine dependence in mice. Heliyon 2024;10:e40584. 10.1016/j.heliyon.2024.e40584 39719994 PMC11667026

[41] Rodríguez-Peña AB, Grande MT, Eleno N et al. Activation of Erk1/2 and Akt following unilateral ureteral obstruction. Kidney Int 2008;74:196–209. 18449171 10.1038/ki.2008.160

[42] Tamouza H, Chemouny JM, Raskova Kafkova L et al. The IgA1 immune complex-mediated activation of the MAPK/ERK kinase pathway in mesangial cells is associated with glomerular damage in IgA nephropathy. Kidney Int 2012;82:1284–96. 10.1038/ki.2012.192 22951891 PMC5177564

[43] Fu S, Zhou Y, Hu C et al. Network pharmacology and molecular docking technology-based predictive study of the active ingredients and potential targets of rhubarb for the treatment of diabetic nephropathy. BMC Complement Med Ther 2022;22:210. 10.1186/s12906-022-03662-6 35932042 PMC9356435

[44] Zhang X, Gu S, Shen S et al. Identification of circular RNA profiles in the liver of diet-induced obese mice and construction of the ceRNA network. Genes (Basel) 2023;14:688. 10.3390/genes14030688 PMC10048691 36980960

[45] Hirata Y, Takahashi M, Kudoh Y et al. Fatty acids promote proinflammatory signaling and cell death by stimulating the apoptosis signal-regulating kinase 1 (ASK1)-p38 pathway. J Biol Chem 2017;292:8174–85. 10.1074/jbc.M116.771519 28360100 PMC5437226

[46] Natarajan SK, Ingham SA, Mohr AM et al. Saturated free fatty acids induce cholangiocyte lipoapoptosis. Hepatology 2014;60:1942–56. 10.1002/hep.27175 24753158 PMC4553418

[47] Kadotani A, Tsuchiya Y, Hatakeyama H et al. Different impacts of saturated and unsaturated free fatty acids on COX-2 expression in C(2)C(12) myotubes. Am J Physiol Endocrinol Metab 2009;297:E1291–303. 10.1152/ajpendo.00293.2009 19755671

[48] Coulthard LR, White DE, Jones DL et al. p38(MAPK): stress responses from molecular mechanisms to therapeutics. Trends Mol Med 2009;15:369–79. 10.1016/j.molmed.2009.06.005 PMC3016890 19665431

[49] Liu ZY, Zhong QW, Tian CN et al. NMDA receptor-driven calcium influx promotes ischemic human cardiomyocyte apoptosis through a p38 MAPK-mediated mechanism. J Cell Biochem 2019;120:4872–82. 10.1002/jcb.27702 30614047

[50] Xiao L, Hu C, Feng C et al. Switching of N-methyl-D-aspartate (NMDA) receptor-favorite intracellular signal pathways from ERK1/2 protein to p38 mitogen-activated protein kinase leads to developmental changes in NMDA neurotoxicity. J Biol Chem 2011;286:20175–93. 10.1074/jbc.M110.188854 21474451 PMC3121511

[51] Ahn YM, Oh SW, Kang UG et al. An N-methyl-D-aspartate antagonist, MK-801, preferentially reduces electroconvulsive shock-induced phosphorylation of p38 mitogen-activated protein kinase in the rat hippocampus. Neurosci Lett 2000;296:101–4. 10.1016/S0304-3940(00)01632-3 11108991

