# Supplementary material for: N-methyl-D-aspartate receptor inhibition protects against obesity-induced kidney disease
Source: Nephrol Dial Transplant. 2026 Feb 11;41(8):1529–42. doi: 10.1093/ndt/gfag027 (PMC13423822; doi:10.1093/ndt/gfag027)
Supplement: gfag027_Supplemental_File [file gfag027_supplemental_file.docx]

**SUPPLEMENTARY MATERIAL**

Supplementary Material and Methods

Supplementary Table 1: Assays for quantitative real-time PCR

Supplementary Table 2: Primary antibodies for Western blot

Supplementary Table 3: Characteristics of patients

Supplementary Table 4: Quantification of immunoperoxidase staining for GluN1, MCP1 and Icam-1 in human kidney biopsies.

Supplementary Figure S1. FFAs treatment enhances Kim1 and FFAR4 expression in HK-2 cells in vitro.

Supplementary Figure S2. GluN1 localization in HK-2 cells upon FFA treatment assessed by confocal z-stacks.

Supplementary Figure S3. Effects of NMDAR antagonists on cell viability.

Supplementary Figure S4. Effects of lower doses of NMDAR antagonists on cell lipid accumulation.

Supplementary Figure S5. GluN1 knockdown is associated with increased inflammatory markers and reduced antioxidant markers in vitro.

Supplementary References

**Supplementary Material and Methods**

**Cell culture and treatments**

HK-2 cells (human renal proximal tubular epithelial cells) (ATCC® CRL-2190™) were maintained in a 1:1 mixture of DMEM (11880, Gibco™) and Ham´s F12 (31765, Gibco™) supplemented with HEPES buffer (20 mM), transferrin (5 µg/mL), insulin (5 µg/mL), dexamethasone (5 x 10^-8^ M), T_3_ (10^-9^ M), sodium selenite (60 nM), D-glucose, EGF (10 ng/mL), fetal bovine serum (FBS, 2%), and penicillin/streptomycin (1%, Sigma Aldrich). The deprivation medium contained all components except Ham´s F12 and FBS. In the complete growth medium, the final concentrations of L-glutamine and glycine in the 1:1 DMEM/Ham’s F12 mixture were 0.5 mM and 0.25 mM, respectively, while in the deprivation medium, L-glutamine and glycine concentrations were 0 mM and 0.4 mM, respectively.

Before treatments, cells were growth arrested in serum-free medium for 5 hours. To induce fat overload in vitro, cells were exposed to different concentrations of long fatty acid mixture (FFAs) of oleate (O7501, Sigma-Aldrich) and palmitate (P9767, Sigma-Aldrich) (2:1 ratio) for 24 hours. Treatments were made in deprivation medium supplemented with 1% fatty acids-free Bovine Serum Albumin (FFA-BSA) (A8806, Sigma-Aldrich). As needed, cells were exposed to a combined treatment of FFAs + NMDAR antagonists such as memantine hydrochloride (0.5 mM, M9292, Sigma-Aldrich) and (+)-MK-801 maleate (0.5 mM, 0924, Tocris Bioscience), or SB203580 inhibitor (20 µM, S1076, Selleckchem), depending on the experimental design.

**Lentiviral production and infection of HK-2 cells**

To overexpress GluN1 in HK-2 cells, we used MISSION ORF NR1 (TRCN0000487776) plasmid and the corresponding control (MISSION TRC3 ORF GFP), both purchased from Sigma-Aldrich. To downregulate GluN1, the shRNA construct was generated by annealing complementary 60-mer oligonucleotides containing the 21-nucleotide target sequence (sense/antisense) separated by a 9-nt spacer. Targeted specificity was confirmed by BLAST. Annealed oligonucleotides were cloned into the AgeI-BamHI site of a U6-driven lentiviral shRNA vector containing Venus GFP under the SV40 promoter to monitor transduction efficiency, as previously reported [1].

To produce lentiviral particles, we used 293T cells co-transfected by the polyethylenimine (PEI) transfection method with the virion packaging plasmids (VSV-G and Δ8.9) and the plasmid overexpressing GluN1 (ORF-NR1) or control vector (ORF-GFP). 293T cells were maintained for 72 hours in DMEM supplemented with 10% FBS, sodium pyruvate, nonessential amino acids, penicillin, and streptomycin to allow production of lentiviral particles. After 72 hours, medium was harvested and centrifuged at 3000 rpm for 10 minutes. Collected supernatant was subsequently filtered through Sartorius Vivaspin filters at 4000 rpm for 1 hour, 4°C. Filtered supernatant was added to HK-2 cells and incubated overnight. Next morning, fresh growth medium was replaced, and cells were left to grow for additional 48 hours before starting puromycin selection. The stable cell line was selected using puromycin selectable marker (2.5 µg/ml, A11138-03, Gibco). Cells were subcultured as required during puromycin selection. The successful overexpression of GluN1 was assessed by Western Blot analysis.

**Viability assay**

To determine the adequate dose of NMDAR antagonists for our experiments, we assessed cell viability using MTT (3-[4,5-dimethylthiazol-2-yl]-2,5-diphenyltetrazolium bromide, M2128, Sigma) assay. HK-2 cells were seeded with different concentrations of Memantine and MK-801 (0.05 mM, 0.1 mM, and 0.5 mM), or combined treatment of fatty acids (0.5 mM) and antagonists for 24 hours. After incubation, treatments were discarded and 100 μl MTT (1 mg/ml) was added to each well and cells were incubated at 37°C, 5% CO_2_ and 95% air for 2 hours. After incubation, MTT solution was aspirated and 100 µl/well of isopropanol was added for 10 minutes at RT until complete dissolution of MTT formazan crystals. Absorbance was measured at 570 nm (L1) and 655 nm (L2) using the BioTek Epoch Microplate Spectrophotometer (Agilent) and calculated by L1-L2.

**Oil red O staining *in vitro***

After FFAs incubation *in vitro*, cells were washed 2x with PBS and fixed with 4% paraformaldehyde for 30 min at room temperature (RT). After rinsing with PBS and 60% Isopropanol (IPA), cells were incubated with 0.6% Oil-Red-O (O0625, Sigma) (in 60% IPA) solution for 10 min at RT, then washed with water to remove residual stain. Cells were distained using 100% IPA, 15 min at RT and the optical density of the solution was measured at 492 nm using the BioTek Epoch Microplate Spectrophotometer (Agilent).

**Animals and experimental protocol**

All animal studies received approval from the University of Lleida´s local Animal Ethics Committee (CEEA 05-01/18), and adhered to all applicable ethical standards and the European Research Council´s guidelines for the care and use of laboratory animals. To establish a mouse model of OIKD, we used C57BL/6J strain, highly susceptible to obesity and OIKD when fed a high-fat diet, that closely mirrors the systemic and renal alterations seen in human disease. Male C57BL/6J mice were purchased from Charles River (Barcelona, Spain), and were housed and maintained in a barrier facility under controlled temperature of 22°C in 12 h-light/dark cycle with ad libitum access to food and water. Pathogen-free procedures were carried on in all mouse rooms. At 12 weeks of age, mice were randomly divided and fed either a standard diet (STD; n=9) (containing 13 kcal% fat and 20 kcal% proteins; Harlan Teklad, Madison, WI, USA) or a high fat diet (HFD; n=27) (containing 60 kcal% fat, 20 kcal% carbohydrate, 20 kcal% protein (D12492, E15742-34, SSNIFF, Soest, Germany). Four weeks after the beginning of the diet regimen, HFD-fed mice were randomly divided into three groups and injected intraperitoneally (IP) with either saline (0,9% NaCl; HFD group; n=9), Memantine (0.2 mg/kg/day; HFD+memantine group; n=9) or MK-801 (0.25 mg/kg/day; HFD+MK-801 group; n=9), three times per week. The dose of memantine used in our study was consistent with that previously reported [2], while the MK-801 dose was reduced by 50% to avoid potential side effects [3, 4], with no behavioural changes observed. Diet feeding and injections were continued for additional 6 weeks. Body weight was measured every week throughout the whole experiment. Individual food intake was measured at three time points during the experiment. Mice were euthanized at 22 weeks of age. Blood was collected by cardiac puncture after an overnight fast, and the animals were perfused with PBS through a puncture in the left ventricle. The organs of interest were collected for histologic examination and molecular analysis. One part of the kidney was fixed in 4% paraformaldehyde/PBS followed by embedding in paraffin and/or Bright Cryo-M-Bed compound (Bright Instrument Co., Huntingdon, UK) for consequent histological and immunohistochemical analysis. The remaining kidney tissue was snap-frozen in liquid nitrogen and kept at −80°C for protein and RNA extractions.

**Patients and human kidney samples**

The study involving human samples were approved by the Ethics Committee for Clinical Investigation at University Hospital Arnau de Vilanova in Lleida (CEIC-2367), ensuring adherence to ethical standards and the Declaration of Helsinki guidelines. All patients provided informed consent before participating, allowing their kidney tissues and medical data to be used for research purposes.

Kidney samples were obtained from diagnostic renal biopsies of obese patients with obesity-related glomerulopathy with proteinuria (OBE; n=5), and patients with focal segmental glomerulosclerosis (FSGS; n=9) who exhibited elevated circulating lipid levels (total cholesterol, LDL, and triglycerides). Human renal biopsies were collected at the University Hospital Arnau de Vilanova in Lleida between 2013 and 2021, with the support of the IRBLleida Biobank (B.0000682) and Biobank and Biomodels Platform ISCIII PT23/00032.

**RNA purification and quantitative real-time PCR**

Total RNA was extracted from cultured cells or whole kidney tissue using the TRIzol reagent (Molecular Research Center, Inc), following the manufacturer´s instructions. Reverse transcription was performed using the First Strand cDNA Synthesis Kit (Applied Biosystems) according to the manufacturer’s instructions. Quantitative real-time PCR (qPCR) with gene-specific probes was performed with a CFX Real-Time PCR detection system (Bio-Rad Laboratories, Madrid, Spain) using TaqMan Universal PCR Master Mix, No AmpErase UNG. Forty cycles at 95°C for 15 seconds and 60°C for 1 minute were performed [5]. Relative mRNA levels were calculated by standard formulae (ΔΔCt method) using GAPDH or TBP as an endogenous control. The results referred to a randomly selected basal sample considered as value = 1.0. Gene-specific probes used in this study are indicated in Supplementary Table 1.

**Protein extraction and western blot analysis**

After desired incubations, cell monolayers were rinsed twice with ice-cold PBS. Cells were scraped and lysed with lysis buffer containing 20 mM Tris (pH7.5), 120 mM NaCl, 0.5% Nonident P-40 (Igepal CA-630), 1 mM EDTA, and 10% Glycerol supplemented 1 mM PMSF, 1 mM Na_3_VO_4_ and protease inhibitor cocktail. Cell extracts were centrifuged at 13.000 rpm for 20 minutes, 4ºC. Supernatant was saved as cytosolic fraction. Pellet was resuspended in a buffer containing 125 mM Tris (pH 6.8) and 2% SDS supplemented with PIC, PMSF and Na_3_VO_4_ and was sonicated 3 times for the interval of 6 sec. Obtained cell lysates (membrane-enriched fraction) were used for the detection of NMDAR1, while the cytosolic fraction was used for the detection of other proteins in the study. Proteins from the kidney cortex were extracted using Triton soluble buffer (50 mM Tris-HCl (pH 7.5), 150 mM NaCl, 1% Triton X-100 and 0.5 µM EDTA supplemented with 1 mM PMSF, 1 mM Na_3_VO_4_ and protease inhibitor cocktail) upon homogenization with TissueLyser LT (Qiagen, Hilden, Germany) (50 Hz, 30 sec, 3 cycles). Afterwards, lysates were incubated on an orbital shaker (Selecta) for 1 hour at 4ºC, and subsequently centrifuged at 13.000 rpm for 15 min at 4ºC. Supernatants were collected and saved at -80ºC. Pellets were resuspended in SDS soluble buffer (50 mM Tris (pH 7.5), 150 mM NaCl, 1% Triton X-100, 0.5 µM EDTA, 2% SDS, 1% Na-Deoxycholate and 1% Igepal CA-360 supplemented with 1 mM PMSF, 1 mM Na_3_VO_4_ and protease inhibitor cocktail) and sonicated 3 times, 6 sec each. After centrifugation at 13.000 rpm, 10 min, 4ºC, supernatants (SDS soluble fraction) were saved and used for the detection of NMDAR1 and Icam1. Concentration of proteins was determined using a DC protein assay kit (Bio-Rad). 20 μg (or 40 µg for NMDAR1 detection) of proteins were electrophoresed on 8%, 10%, 12% or 15% SDS-PAGE gels, as appropriate, and transferred to PVDF membrane (pore size 0.45 μm, Immobilon-P, Millipore). Membranes were blocked for 1 hour with 5% skim milk in Tris-buffer saline containing 0.1% Tween-20 (TBST) and subsequently probed with primary antibody (Supplementary Table 2) overnight at 4ºC. After primary antibody incubation, membranes were washed and incubated with corresponding horseradish peroxidase-conjugated secondary antibodies (Supplementary Table 2) at 1/10.000 for 1 hour, RT. The immunoreaction was visualized using chemiluminescent kits EZ ECL (Biological Industries) or ECL Advanced (Amersham Biosciences). Images were digitally acquired by ChemiDoc™ MP Imaging System (Bio-Rad). Positive immunoreactive bands were quantified by densitometry and compared with the expression of adequate loading control.

**Protein nuclear extraction**

HK-2 cells were exposed to FFAs and/or a combined treatment of FFAs + NMDAR antagonists for 60 minutes, depending of the experimental design. Cell monolayer was rinsed twice with ice-cold PBS and scraped in Hypotonic buffer (20 mM Tris-HCl, pH 7.4, 10 mM NaCl, 3 mM MgCl_2_, 1 mM PMSF and protease inhibitor cocktail). After 15 minutes of incubation on ice, 10 μl of 10% Igepal/200 μl lysate was added and vortexed for 10 seconds at the highest setting. Homogenate was centrifuged at 10.000 x g for 40 minutes at 4°C and supernatant containing cytoplasmic fraction was stored at -80°C. Remaining cell pellet was further resuspended in Cell Extraction buffer (100 mM Tris, pH 7.4, 100 mM NaCl, 1% Triton X-100, 1 mM EDTA, 10% glycerol, 1 mM EGTA, 0.1% SDS, 0.5% deoxycholate, 20 mM Na_4_P_2_O_7_, 2 mM Na_3_VO_4_, 1 mM NaF, 1 mM PMSF and protease inhibitor cocktail) and incubated on ice for 30 minutes, vortexing every 10 minutes. Obtained cell extract was centrifuged for 30 minutes at 14.000 x g, 4°C and supernatant containing nuclear fraction was stored at -80°C.

**Histopathologic analysis and immunohistochemistry**

For histological analysis, paraffin-embedded kidney sections (5 µm thick) were stained using the periodic acid-Schiff (PAS) technique. The stained sections were then examined by two independent researchers according to pre-defined criteria. Kidney injury (kidney injury score) was evaluated by scoring the loss of brush border and cytoplasmic vacuolation in renal tubule epithelium. The scoring of 5 to 10 randomly selected, non-overlapping fields (10× original magnification) of each sample was done using a 5-point scale, as described previously [6, 7]: 0 = no damage, 1 = 1–10% of damaged kidney cortex, 2 = 10–25% damage, 3 = 25–50% damage, 4 = 50–75% damage, 5 = more than 75% damage. The reliability of such scores for the interpretation of renal damage has already been reported [6, 7].

To assess renal lipid content, Bright Cryo-M-Bed-embedded frozen kidney sections (8 µm thick) were fixed with 4% Paraformaldehide/PBS 15 min at RT. After washing with PBS, sections were stained with 0.6% Oil-Red O solution (in 60% IPA) for 10 min at RT and counterstained with Haematoxylin (MAD-108.1000, Germany) for 1 min. Finally, sections were mounted with Fluoromount-G Southern Biotech 0100-01 and examined using an Olympus BX50 microscope with an Olympus automatic camera system. The stained area for each section was determined applying colour thresholding and measuring area fractions with ImageJ software 1.53k (NIH Public Domain, RRID: SCR_003070).

Immunostaining for F4/80, NMDAR1, Icam1 and MCP1 was carried out on 5 µm thick paraffin-embedded kidney sections that were deparaffinized through xylene and rehydrated through graded ethanol concentrations (100%, 95% and 75%) and distilled water. Antigen retrieval was done by incubating kidney sections with Proteinase K (20 µg/ml in TE buffer containing 50 mM Tris base and 1 mM EDTA, pH 8.0) for 15 minutes (for F4/80 staining), or boiling in 10 mM citrate buffer (pH6) for 10 min (for NMDAR1, Icam1, MCP1 staining). Endogenous peroxidase quenching (30 min incubation in 0.6% (w/v) H_2_O_2_/PBS) was followed by blocking of non-specific binding with 5% normal goat serum in 5%BSA/PBS (for F4/80; 1 hour, RT) or 2.5% normal horse serum (NHS) (anti-rabbit ImmPRESS MP-6401 or anti-mouse ImmPRESS MP-7402, Vector) (for NMDAR1, Icam1, MCP1; 20 min, RT). Primary antibodies for the detection of F4/80 (#MCA497R, BioRad; 1/50), NMDAR1 (#PA3-102, Invitrogen; 1/500), Icam1 (sc-8439, Santa Cruz; 1/500), and MCP1 (ab25124, Abcam; 1/500) were incubated overnight at 4ºC. After washing with PBS, slides were treated with the corresponding ready-to-use ImmPRESS IgG Polymer Reagent (anti-rabbit MP-6401 or anti-mouse MP-7402) for 30 min (for NMDAR1, Icam1, MCP1), or with avidin-biotin-peroxidase complex (ABC) [8] (for F4/80) (Vector Laboratories, Inc. USA). Subsequently, sections were incubated with 3,3’- diaminobenzidine as the chromogen (Vector Laboratories, Inc. USA) and counterstained with haematoxylin for 1 min, RT. Negative controls were performed by incubation with non-specific immunoglobulin of the same isotype as the primary antibody but with the omission of the primary antibody. After immunostaining, slides were dehydrated, cleared in xylene and mounted with DPX permanent mounting medium. Stained tissue sections were examined using an Olympus BX50 microscope with an Olympus automatic camera system.

**Biochemical measurements**

Serum cholesterol, LDL and triglycerides were assessed using conventional clinical procedures with a multichannel Hitachi Modular analyser (Roche Diagnostics, Indianapolis, IN, USA). Glucose levels in the serum were measured using the commercially available Glucose-TR kit (ref. 1001190, Spinreact, Barcelona, Spain). IL-6 from the cell supernatant was determined using the ELISA kit (950.030.096, Diaclone).

**Immunofluorescence** **staining**

For immunofluorescence analysis of NMDAR in cells, HK-2 culture were grown on coverslips, as previously described. After desired incubation, cells were rinsed twice with PBS and fixed with 4% Paraformaldehyde/PBS for 10 min, RT. After permeabilization with 0.1% Triton X-100/PBS for 10 min, cells were incubated with 1%BSA/PBS to block the non-specific binding for 1 hour, RT. Primary anti-rabbit antibody for NMDAR1 (#PA3-102, Invitrogen; 1/200) was incubated overnight at 4ºC. Cell monolayer was rinsed with PBS and incubated with goat anti-rabbit Alexa 488 (A-11008, Invitrogen; 1/500) secondary antibody and Alexa Fluor Phalloidin 568 (Invitrogen, A12380, Invitrogen; 1/80) for 1 hour, RT. Nuclear counterstaining was performed with Hoechst H33258 (Sigma) for 10 minutes, RT.

Paraformaldehyde-fixed mouse kidneys were immersed in 30% sucrose overnight and embedded in Bright Cryo-M-Bed compound for freezing. Frozen kidney sections (8 µm thick) were permeabilized with 0.1% Triton/PBS for 15 min, washed with PBS and incubated with Carbo Free Blocking solution (SP 5040, Vector) for 30 min. Blocking of non-specific binding was done with 10% normal goat serum in 1% BSA/TBS for 1 hour, RT, and sections were incubated with anti-rabbit NMDAR1 (#PA3-102, Invitrogen; 1/200) and LTA (B-1325, Vector Laboratories; 1/400) overnight at 4ºC. After rinsing with TBS+0.025% Triton-X100, sections were incubated with goat anti-rabbit Alexa 488 secondary antibody (A-11008, Invitrogen; 1/500, visualization of NMDAR1) and Streptavidin Alexa Fluor 594 Conjugate (S32356, Invitrogen; 1/2000) (visualization of LTA) for 1 hour at RT. Nuclei were counterstained with Hoechst H33258 (Sigma-Aldrich), and sections were mounted with Fluoromount-G (0100-01, Southern Biotech). Stained cells and kidney sections were examined using an Olympus FV1000 confocal laser-scanning microscope with a Digital Camera System.

**Supplementary Table 1: Assays for quantitative real-time PCR**

| **Gene** | **Organism** | **Reference** | **Vendor** |
| --- | --- | --- | --- |
| TBP | Mouse | Mm00446971_m1 | Applied Biosystems |
| ICAM1 | Mouse | Mm00516023_m1 | Applied Biosystems |
| NMDAR1 | Mouse | Mm00433790_m1 | Applied Biosystems |
| IL6 | Mouse | Mm00446190_m1 | Applied Biosystems |
| MCP1 | Mouse | Mm00441242_m1 | Applied Biosystems |
| IL1β | Mouse | Mm00434228_m1 | Applied Biosystems |
| NQO-1 | Mouse | Mm.PT.58.10871473 | Integrated DNA Technologies |
| GAPDH | Human | Hs99999905-m1 | Applied Biosystems |
| MCP1 | Human | Hs00234140_m1 | Applied Biosystems |
| NMDAR1 | Human | Hs00609557_m1 | Applied Biosystems |
| IL1α | Human | Hs00174092_m1 | Applied Biosystems |
| IP-10 | Human | Hs00171042_m1 | Applied Biosystems |
| IL18 | Human | Hs01038788_m1 | Applied Biosystems |
| IL-6 | Human | Hs.PT.58.40226675 | Integrated DNA Technologies |
| ICAM1 | Human | Hs.PT.58.4746364 | Integrated DNA Technologies |
| Gclc | Human | Hs.PT.58.2967824 | Integrated DNA Technologies |
| Nrf2 | Human | Hs.PT.58.28159373 | Integrated DNA Technologies |
| HO-1 | Human | Hs.PT.58.45340055 | Integrated DNA Technologies |
| NQO1 | Human | Hs.PT.58.2697277 | Integrated DNA Technologies |

**Supplementary Table 2: Primary antibodies for Western blot**

| **Antibody** | **ID number** | **Vendor** | **Dilution** |
| --- | --- | --- | --- |
| NMDAR1 | #556308 | Pharmingen | 1/500 |
| α-Tubulin | #T5168 | Sigma | 1/5000 |
| Icam1 | sc-8439 | Santa Cruz | 1/1000 |
| HO-1 | sc-136960 | Santa Cruz | 1/500 |
| Gclc | sc-390811 | Santa Cruz | 1/500 |
| IL6 | sc-32296 | Santa Cruz | 1/200 |
| GAPDH | #ab8245 | Abcam | 1/10000 |
| NMDAR1 | #32-0500 | Invitrogen | 1/200 |
| p-Akt (Ser 473) | #4060S | Cell Signalling | 1/2000 |
| p- Erk1/2 (Thr 202/Tyr 204) | #675502 | BioLegend | 1/5000 |
| p-38 (Tyr 182) E-1 | sc-166182 | Santa Cruz | 1/1000 |
| Total Erk1/2 | #686902 | BioLegend | 1/1000 |
| Total p38 | #622401 | BioLegend | 1/1000 |
| p-MKK3 (Ser 189)/MKK6 (Ser 207) | #12280 | Cell Signalling | 1/1000 |
| Nrf2 | sc-365949 | Santa Cruz | 1/500 |
| Flotilin | #610820 | BD Transduction | 1/1000 |
| Histone 3 | SAB4500352 | Sigma | 1/1000 |
| MCP1 | ab25124 | Abcam | 1/1000 |
| Peroxidase AffiniPure® Goat Anti-Mouse IgG (H+L) | #115-035-003 | Jackson Immunoresearch | 1/10000 |
| Anti-rabbit IgG, HRP- linked antibody | #7074 | Cell Signaling | 1/10000 |
| Peroxidase AffiniPure® Goat Anti-Rat IgG (H+L) | #112-035-003 | Jackson Immunoresearch | 1/10000 |

**Supplementary Table 3: Characteristics of patients**

|  |  |  |  |  |  |  | | |  |
| --- | --- | --- | --- | --- | --- | --- | --- | --- | --- |
| Number of patients | 14 |  |  |  |  |  | | |  |
| Age (years) | 50,1 ± 13,1 |  |  |  |  |  | | |  |
|  |  |  |  |  |  |  | | |  |
| BMI (kg/m²) | 30,03 ± 10,6 |  |  |  |  |  | | |  |
| Systolic BP (mmHg) | 132,8 ± 19,1 |  |  |  |  |  | | |  |
| Diastolic BP (mmHg) | 78,1 ± 9,2 |  |  |  |  |  | | |  |
| Total proteins (g/dL) | 6,3 ± 1,0 |  |  |  |  |  | | |  |
| Serum albumin (g/dL) | 3,4 ± 0,8 |  |  |  |  |  | | |  |
| Microalbuminuria (mg/L) | 862,2 ± 831,3 |  |  |  |  |  | | |  |
| eGFR (mL/min/1.73 m²) | 71,2 ± 33,7 |  |  |  |  |  | | |  |
|  |  |  |  |  |  |  | | |  |
| Medical diagnosis | N (%) | BMI (kg/m²) | CHOLE (mg/dL) | LDL (mg/dL) | TG (mg/dL) | Microalbuminuria (mg/L) | | |  |
| OBE | 5 (35,71%) | 42,1 ± 7,5 | 172,2 ± 68,2 | 123,8 ± 52,5 | 162,5 ± 81,9 | 1162,3 ± 1253,9 | | |  |
| FSGS | 9 (64,29%) | 23,31 ± 3,7 | 310,6 ± 176,9 | 195,8 ± 114,1 | 296,2 ± 334,3 | 695,5 ± 499,4 | | |  |
|  |  |  |  |  |  | |  |  | |

**Supplementary Table 3.** **Characteristics of patients**. Data are shown as mean ± standard deviation or as number of cases (%). BMI, body mass index; BP, blood pressure; eGFR, estimated glomerular filtration rate; OBE, patients with obesity and proteinuria; FSGS, focal segmental glomerulosclerosis; CHOLE, total cholesterol; LDL, low-density lipoprotein; TG; triglycerides.

**Supplementary Table 4. Quantification of immunoperoxidase staining for GluN1, MCP1 and Icam-1 in human kidney biopsies.** OBE, patients with obesity-related glomerulopathy with proteinuria; FSGS, patients with focal segmental glomerulosclerosis; Control, kidney sections with no evidence of pathology. Data are presented as mean ± SEM (n=3-4, healthy tissue; n=8-9, FSGS; n=4, OBE). *p=0.0206 Control vs. OBE.

**
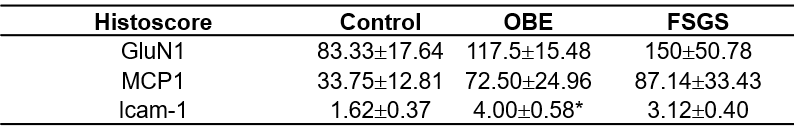
**

**
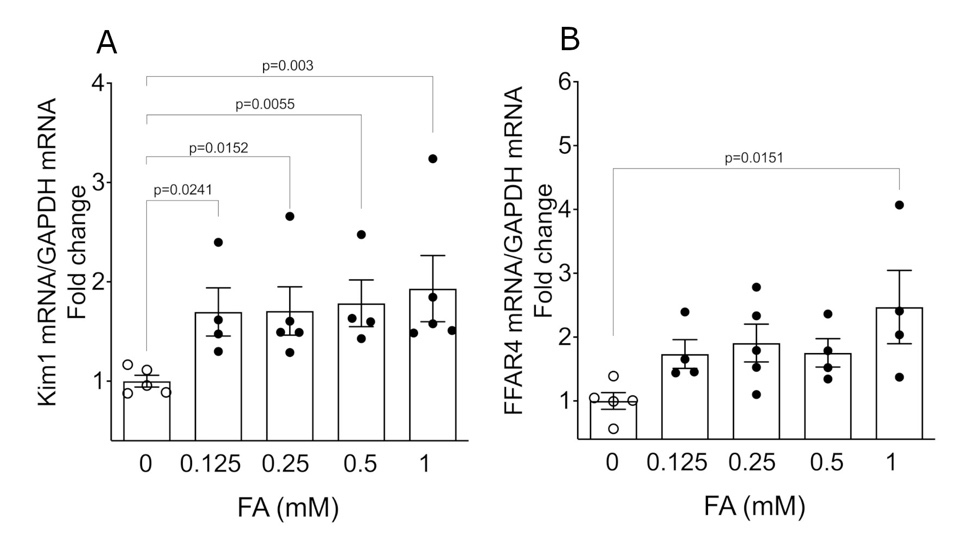
**

**Supplementary Figure S1**. **FFAs treatment enhances Kim1 and FFAR4 expression in HK-2 cells *in vitro*.** Human renal proximal tubular epithelial (HK-2) cells were incubated in serum-free medium or increasing concentrations of FFAs (0,125 mM to 1 mM) (**A-H**) for 24 hours. (**A-B**) Total mRNA was extracted from HK-2 cells and mRNA levels were assessed by quantitative real-time PCR. The relative mRNA levels of Kim1 and FFAR4 were calculated and expressed as fold change over control after normalizing for GAPDH. Data are presented as mean ± SEM of at least n = 2 independent experiments. FFAs – free fatty acids.

**
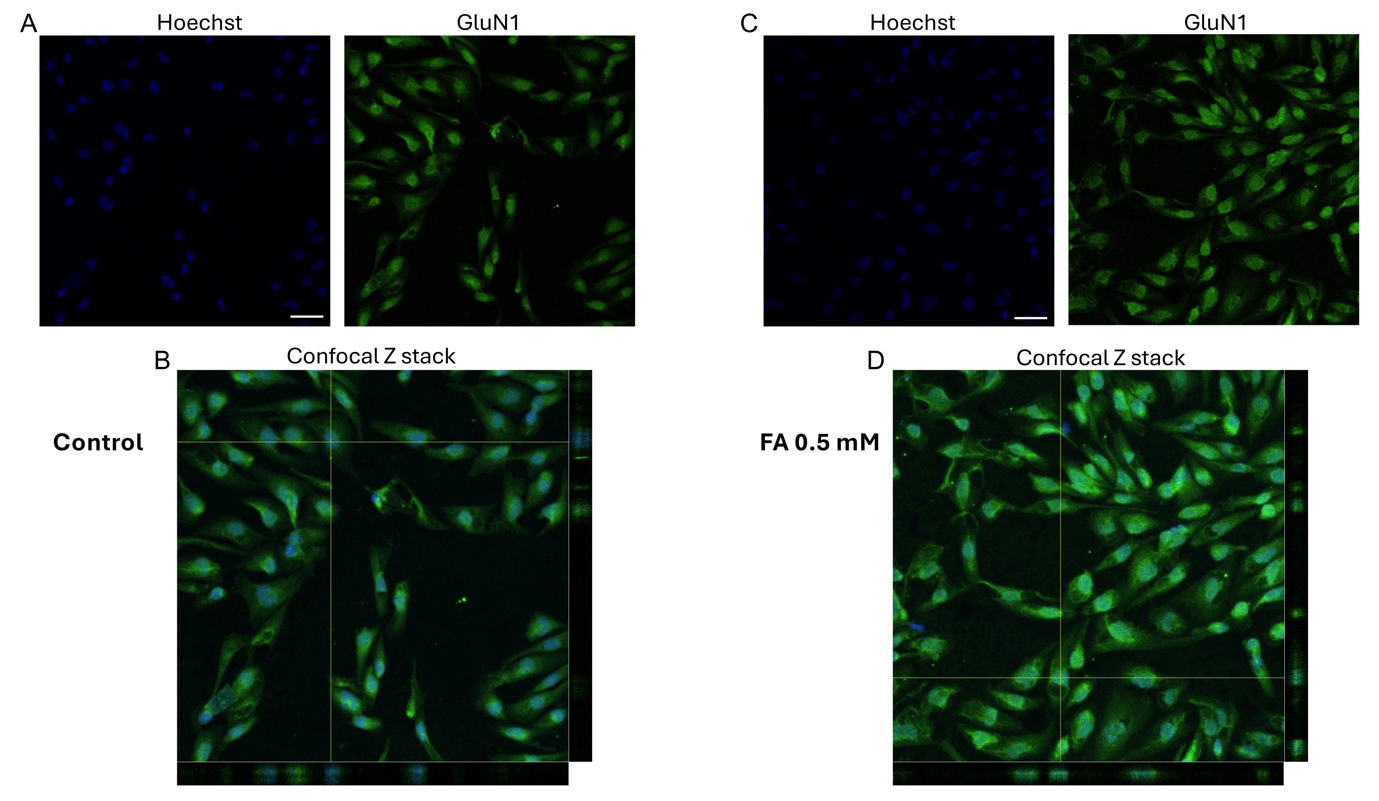
**

**Supplementary Figure S2.** **GluN1 localization in HK-2 cells upon FFA treatment assessed by confocal z-stacks.** Representative confocal images of HK-2 cells stained for GluN1 (green) and Hoechst (blue) under control (a, b) and FA 0.5 mM treatment (c, d). Representative single-plane optical sections (a, c) and orthogonal (XZ/YZ) reconstructions from the full Z-stack (b, d). GluN1 signal is predominantly cytoplasmic/perinuclear. Scale bar, 50 µm.

**
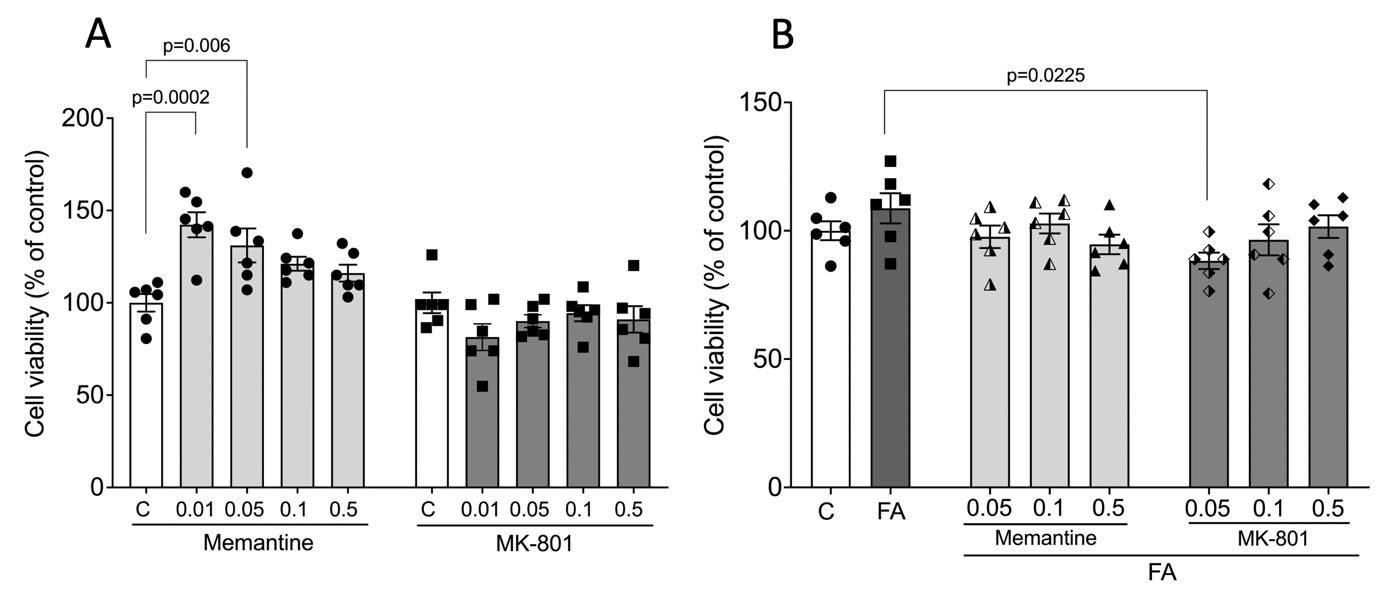
**

**Supplementary Figure S3. Effects of NMDAR antagonists on cell viability**. HK-2 cells were exposed to different concentrations of Memantine and MK-801 (0.01 mM, 0.05 mM, 0.1 mM and 0.5 mM) (**a**), as well as to a combined treatment of FA 0.5 mM and antagonists (0.05 mM, 0.1 mM and 0.5 mM) (**b**). Absorbance was measured at 570 nm and 655 nm, and cell viability was presented as a percentage of control. Data are presented as mean ± SEM of at least n=3 independent experiments.

**
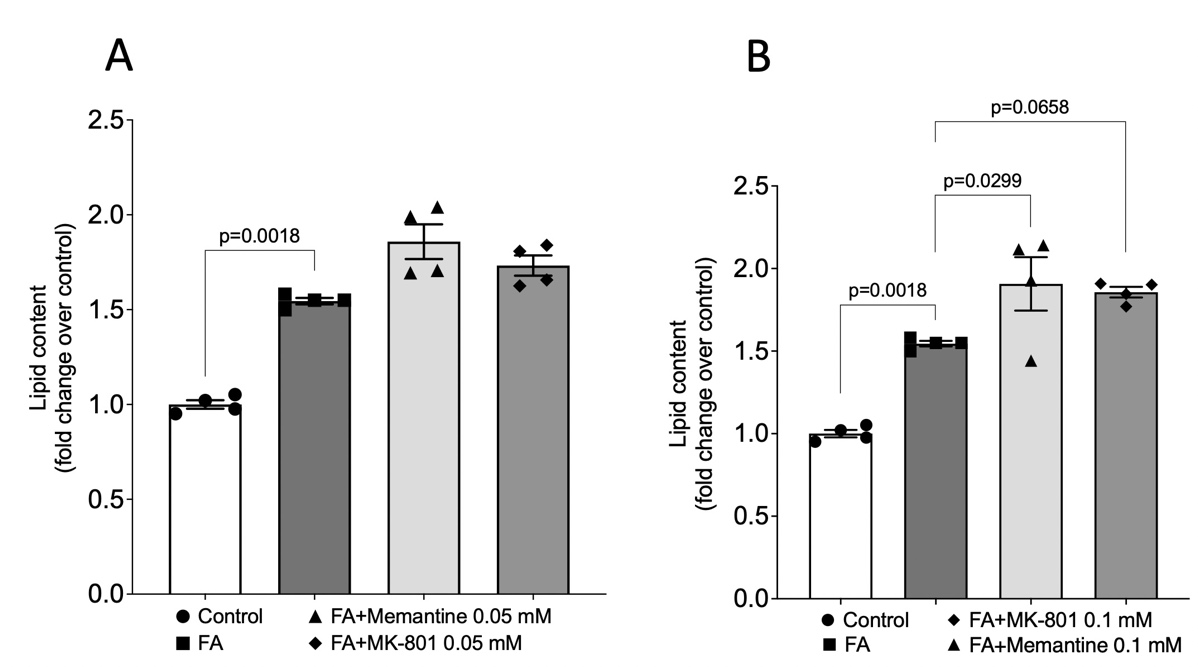
**

**Supplementary Figure S4**. **Effects of lower doses of NMDAR antagonists on cell lipid accumulation**. HK-2 cells were incubated in serum-free medium or treated with a combination of FA 0.5 mM and varying concentrations of antagonists (0.1 mM and 0.05 mM) (**a, b**). Quantification of cellular lipid content was assessed as explained in Methods and data are presented as mean ± SEM of at least n=2 independent experiments.


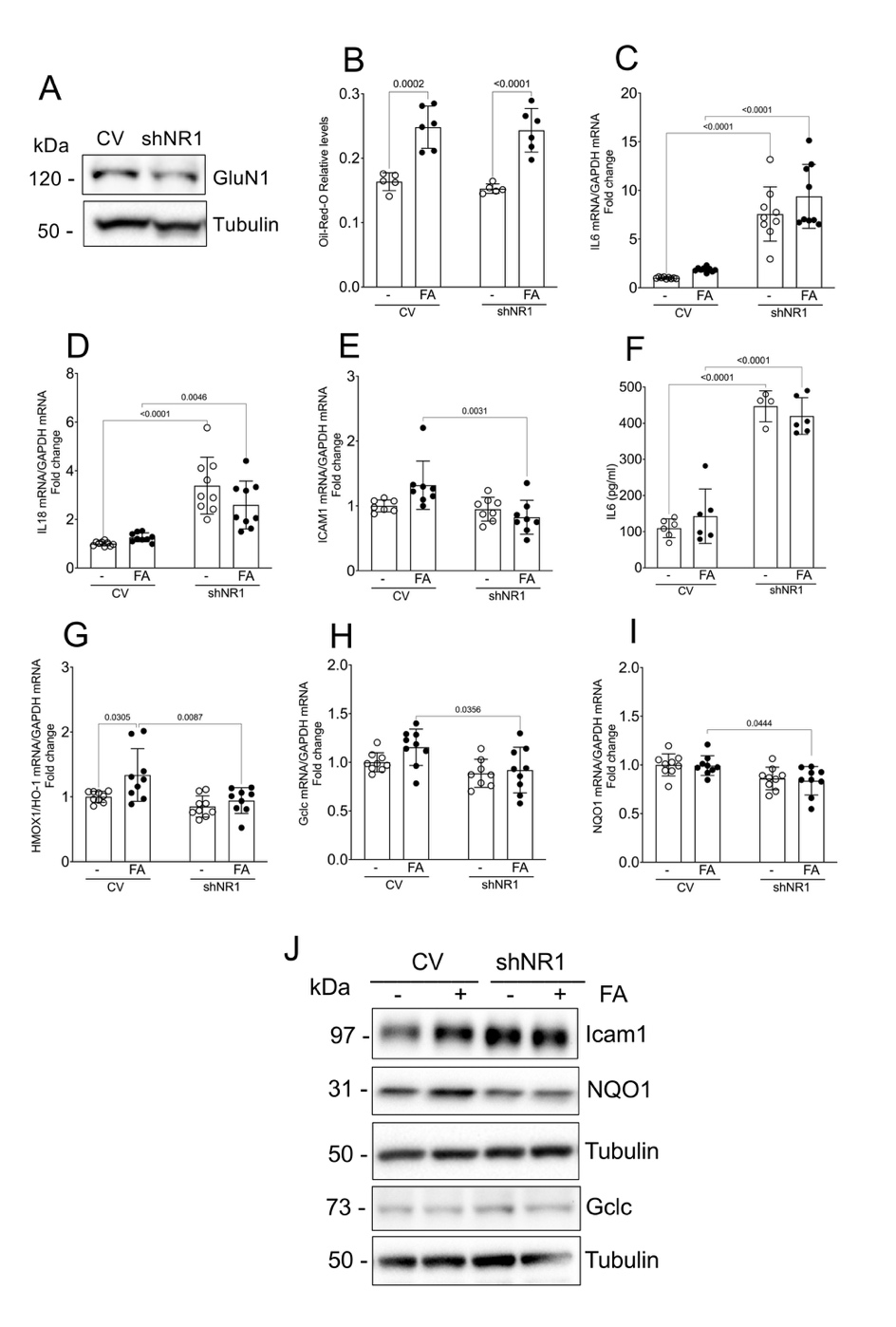


**Supplementary Figure S5**. **GluN1 knockdown is associated with increased inflammatory and reduced antioxidant markers in vitro.** (**A-J**) GluN1 was stably downregulated in HK-2 cells, and the cells were incubated with FFAs (0.5 mM) or left untreated for 24 h. (**B**) Quantification of cellular lipid content in HK-2 cels (Oil Red O). Data are presented as mean ± SEM of at least n = 2 independent experiments. (**C-E**; **G-I**) Total mRNA was extracted from HK-2 cells and mRNA levels were assessed by quantitative real-time PCR. The relative mRNA levels of IL-6, IL18, Icam-1, HO-1, Gclc, and NQO1 were calculated and expressed as fold change over control after normalizing for GAPDH. Data are presented as mean ± SEM of at least n = 3 independent experiments. (**F**) Measurement of IL6 secretion into the medium using an ELISA assay. (**J**) Cell lysates were immunoblotted with antibodies against Icam-1, NQO1, and Gclc. The same samples were reprobed with antibodies against tubulin to ensure equal loading. (**J**) Representative Western blots. CV - control vector; shNR1 - cells with downregulated GluN1 subunit. FFAs – free fatty acids.

**Supplementary References**

1. Bozic M, de Rooij J, Parisi E*, et al.* Glutamatergic signaling maintains the epithelial phenotype of proximal tubular cells. J Am Soc Nephrol 2011;22(6):1099-1111

2. Bassil N, Thaipisuttikul P, Grossberg GT. Memantine ER, a once-daily formulation for the treatment of Alzheimer's disease. Expert Opin Pharmacother 2010;11(10):1765-1771

3. Liu JL, Li M, Dang XR*, et al.* A NMDA receptor antagonist, MK-801 impairs consolidating extinction of auditory conditioned fear responses in a Pavlovian model. PLoS One 2009;4(10):e7548

4. Chen HS, Lipton SA. The chemical biology of clinically tolerated NMDA receptor antagonists. J Neurochem 2006;97(6):1611-1626

5. Bozic M, Caus M, Rodrigues-Diez RR*, et al.* Protective role of renal proximal tubular alpha-synuclein in the pathogenesis of kidney fibrosis. Nat Commun 2020;11(1):1943

6. Eritja À, Caus M, Belmonte T*, et al.* microRNA Expression Profile in Obesity-Induced Kidney Disease Driven by High-Fat Diet in Mice. Nutrients 2024;16(5)

7. Potočnjak I, Domitrović R. Carvacrol attenuates acute kidney injury induced by cisplatin through suppression of ERK and PI3K/Akt activation. Food Chem Toxicol 2016;98(Pt B):251-261

8. Vićovac L, Bozić M, Bojić-Trbojević Z*, et al.* Carcinoembryonic antigen and related molecules in normal and transformed trophoblast. Placenta 2007;28(2-3):85-96
